# A review on bacteria-derived antioxidant metabolites: their production, purification, characterization, potential applications, and limitations

**DOI:** 10.1007/s12272-025-01541-5

**Published:** 2025-04-10

**Authors:** Nazli Pinar Arslan, Fakhrul Azad, Tugba Orak, Aysenur Budak-Savas, Serkan Ortucu, Pranav Dawar, Mustafa Ozkan Baltaci, Hakan Ozkan, Nevzat Esim, Mesut Taskin

**Affiliations:** 1 Vocational School of Health Services, Bingol University, Bingol, Turkey; 2 Department of Biochemistry and Cell Biology, Chobanian and Avedisian School of Medicine, Boston University, Boston, MA USA; 3 Department of Molecular Biology and Genetics, Science Faculty, Ataturk University, 25240 Erzurum, Turkey; 4 Department of Medical Pharmacology, Faculty of Medicine, Ataturk University, Erzurum, Turkey; 5 Department of Molecular Biology and Genetics, Science Faculty, Erzurum Technical University, Erzurum, Turkey; 6 Environmental Molecular Sciences Laboratory (EMSL), Pacific Northwest National Laboratory (PNNL), Richland, WA USA; 7 Department of Molecular Biology and Genetics, Science and Art Faculty, Bingol University, Bingol, Turkey

**Keywords:** *Actinobacteria*, *Cyanobacteria*, Exogenous antioxidants, Natural metabolites, Oxidative stress, *Proteobacteria*

## Abstract

Antioxidants are organic molecules that scavenge reactive oxygen species (ROS) and reactive nitrogen species (RNS), thereby maintaining cellular redox balance in living organisms. The human body synthesizes endogenous antioxidants, whereas humans obtain exogenous antioxidants from other organisms such as plants, animals, fungi, and bacteria. This review primarily focuses on the antioxidant potential of natural metabolites and extracts from five major bacterial phyla, including the well-studied *Actinobacteria* and *Cyanobacteria*, as well as less-studied *Bacteroides*, *Firmicutes*, and *Proteobacteria.* The literature survey revealed that the metabolites and the extracts with antioxidant activity can be obtained from bacterial cells and their culture supernatants. The metabolites with antioxidant activity include pigments, phycobiliproteins, polysaccharides, mycosporins-like amino acids, peptides, phenolic compounds, and alkaloids. Both metabolites and extracts demonstrate in vitro antioxidant capacity through radical-scavenging, metal-reducing, and metal-chelating activity assays. In in vivo models, they can scavenge ROS and RNS directly and/or indirectly eliminate them by enhancing the activities of antioxidant enzymes, such as catalase, superoxide dismutase, and glutathione peroxidase. Due to their antioxidant activities, they may find applications in the cosmetic industry as anti-aging agents for the skin and in medicine as drugs or supplements for combating oxidative stress-related disorders, such as neurodegenerative diseases and diabetes. The literature survey also elucidated that some metabolites and extracts with antioxidant activity also exhibited strong antimicrobial properties. Therefore, we consider that they may have future applications in the treatment of infectious diseases, the preparation of pathogen-free healthy foods, and the extension of food shelf life.

## Introduction

In biological systems, reactive oxygen species (ROS) and reactive nitrogen species (RNS) regulate various cellular processes when present at the appropriate levels from low to mild, however, their excessive accumulation can damage the cellular molecules (nucleic acids, proteins, lipids, and carbohydrates), thereby causing various health disorders (Martinez and Andriantsitohaina 2009; Phaniendra et al. 2015; Jomova et al. 2023). Therefore, keeping ROS and RNS levels at a tolerable level is extremely important for the regular functioning of the cellular processes.

In humans, this balance is primarily achieved through the body’s endogenous antioxidant system, which includes both enzymatic and non-enzymatic antioxidants. However, exogenous antioxidants from natural sources also aid the endogenous antioxidant system in eliminating excess ROS/RNS (Arslan et al. 2023). We obtain exogenous antioxidants mainly from plant sources through diet; however, mushrooms, lichens, micro-and macroalgae, and prokaryotic organisms (the domains *Bacteria* and *Archaea*) can also be an additional source of exogenous antioxidants (Chandra et al. 2020; Vladkova et al. 2022; Arslan et al. 2023; Dost et al. 2024; Esim et al. 2024). Exogenous antioxidants are widely used in the cosmetic industry as anti-aging molecules for the skin (Montero-Lobato et al. 2018; Kageyama et al. 2019; Ávila-Román et al. 2021; Geraldes and Pinto 2021). Furthermore, they exert a protective role against oxidative stress-related health disorders, including neurodegenerative diseases, infertility, diabetes, cardiovascular diseases, Crohn’s disease, rheumatoid arthritis, and inflammatory bowel diseases (Ashok et al. 2022; Martemucci et al. 2022).

Bacteria can synthesize various bioactive compounds, including those with antioxidant activity (Ramasubburayan et al. 2015; Buijs et al. 2019; Rangseekaew and Pathom-Aree 2019; Singh et al. 2020). However, the chemical structure and biological activity of some bacterial antioxidants differ from those of plant and animal origin. For instance, some antioxidant compounds, such as phycobiliproteins, prodigiosin, and violacein, are exclusively synthesized by bacteria, but not by plants or fungi (Sajjad et al. 2018; Cheng et al. 2022; Suphan et al. 2023).

To date, several studies have been published on the antioxidants derived from the bacterial phylum Actinobacteria; however, they have primarily focused on only one group of *Actinobacteria*-derived antioxidant molecules, namely pigments, polysaccharides, peptides, or phenolic compounds. For example, from 2021 to 2024, only three review articles focused on different groups of antioxidant molecules in Actinobacteria (Kamala et al. 2020; Chandra et al. 2020; Rani et al. 2021). Furthermore, in contrast to *Actinobacteria*, there is no review article on exogenous antioxidants derived from other bacterial phyla, namely Bacteroidetes, *Cyanobacteria*, *Firmicutes,* and *Proteobacteria.* Considering the existing knowledge gap, this work aims to assess the literature on antioxidant metabolites obtained from not only the phylum Actinobacteria but also other phyla within the domain *Bacteria*. Additionally, it is worth noting that the current study focused on the various groups of antioxidant molecules, including pigments, polysaccharides, peptides, phycobiliproteins, mycosporine-like amino acids, phenolics, and alkaloids, from these phyla.

## Oxidative stress and antioxidants

In living systems, free radicals are primarily generated from oxygen (ROS) and nitrogen (RNS) (Poljsak et al. 2013). ROS include superoxide (O_2_·^−^), hydroxyl (·OH), alkoxyl (RO·), peroxyl (ROO·), hydroperoxyl (HO_2_·), carbonate (CO_3_·^−^) radicals as well as hydrogen peroxide (H_2_O_2_), singlet oxygen (^1^O_2_), ozone (O_3_) and hypobromous acid (HOBr) radicals. Examples of RNS include nitric oxide (NO·), nitrogen dioxide (NO_2_·), nitrous acid (HNO_2_), nitroxyl anion (NO^−^), nitrosonium cation (NO^+^), and peroxynitrite (ONOO^−^). RNS are formed when NO^·^ interacts with reactive oxygen species, such as O_2_·^−^ and H_2_O_2_ (Khan et al. 2023). The reactivities of RNS are in the following order: NO^·^ < NO_2_^·^ < ONOO^−^. Although NO^·^ is less prone to chemical reactivity, its reaction with O_2_·^−^ generates a highly reactive and toxic species, ONOO − , which damages lipid, protein, and DNA molecules (Martemucci et al. 2022).

ROS and RNS are produced as a result of cellular metabolism in mitochondria, chloroplasts, endoplasmic reticulum, peroxisomes, lysosomes, cytoplasm, and plasma membranes (Jasid et al. 2006; Hameister et al. 2020). However, the electron transport chain (ETC) is considered the primary source of ROS, being solely responsible for the production of approximately 90% of the total cellular ROS. Among the ROS species, the O_2_·^−^ radical is the most abundant and reactive. About 0.2–2.0% of all molecular oxygen exhausted by mitochondria is reduced to O_2_·^−^ radicals, which are then converted to other ROS species, such as H_2_O_2_ and ·OH radicals (Tirichen et al. 2021). Furthermore, various external stimuli, such as ionizing radiation, solar radiation, smoking, foods, drugs, heavy metals, organic solvents, and pesticides can cause the excessive accumulation of cellular ROS and RNS (Hameister et al. 2020; Phaniendra et al. 2015; Ashok et al. 2022; Curieses Andrés et al. 2023).

A normal level of ROS and RNS is essential for the efficient functioning of the cells, where they serve as signaling molecules by regulating various biochemical transformations or modulating signal transduction pathways. Functioning as a cellular signal, they participate in diverse processes, including cell survival, proliferation, migration, differentiation, and apoptosis (Martinez and Andriantsitohaina 2009; Phaniendra et al. 2015; Jomova et al. 2023). On the contrary, the excessive accumulation of endogenous and/or exogenous ROS and RNS damages cellular molecules (nucleic acids, membranes, proteins, carbohydrates, and lipids) and tissues, leading to inflammation and consequent emergence of various health-related issues, such as skin aging, infertility, diabetes mellitus, neurodegenerative disorders (Alzheimer’s and Parkinson’s disease), cardiovascular diseases, respiratory diseases, cataract, rheumatoid arthritis, and cancers (Phaniendra et al. 2015; Eddaikra and Eddaikra 2021; Ashok et al. 2022; Yan et al. 2022; Jomova et al. 2023). Therefore, there is a need for a tight balance between ROS generation and scavenging to maintain cellular ROS homeostasis.

**Fig. 1:**
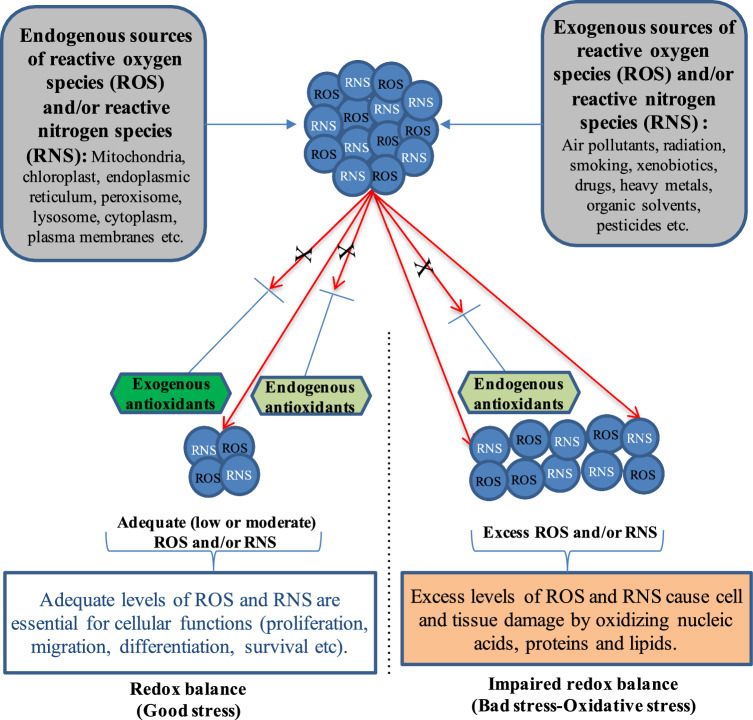
The role of antioxidants against oxidative stress. Reactive oxygen species (ROS) and reactive nitrogen species (RNS) are produced endogenously by various cellular organelles, including mitochondria, chloroplasts, endoplasmic reticulum, lysosomes, cytoplasm, and plasma membranes, as well as by external factors such as ionizing radiation, smoking, drugs, and heavy metals. An adequate level of ROS and RNS, namely a good redox balance, is essential for various cellular mechanisms. On the contrary, the excess accumulation of ROS and RNS, namely oxidative stress, damages cellular molecules (nucleic acids, membranes, proteins, carbohydrates, and lipids) and tissues. In humans, endogenous and exogenous antioxidants scavenge ROS and RNS molecules, thereby maintaining cellular redox homeostasis

Antioxidants are small organic molecules or large enzymes that can scavenge ROS and RNS molecules, thereby maintaining cellular oxidative homeostasis. In animals, including humans, both exogenous and endogenous antioxidants synergistically combat excess ROS/RNS molecules, thereby maintaining cellular ROS homeostasis and alleviating oxidative stress (Rao et al. 2011; Hussain and Kayani 2020) (Fig. 1).

Endogenous antioxidants, synthesized by the human body, include both enzymatic and non-enzymatic antioxidants. The most-well known endogenous enzymatic antioxidants are superoxide dismutase (SOD), catalase (CAT), peroxidase (POX), glutathion reductase (GR), glutathione peroxidase (GPx), paraoxonase (PON) and thioredoxin (Trx) (Esim et al. 2024). Examples of endogenous non-enzymatic antioxidants include melatonin, coenzyme Q10, bilirubin, albumin, ferritin, myoglobin, transferrin, glutathione, uric acid, and polyamines (Arslan et al. 2023).

The best-known exogenous antioxidants are vitamin E, vitamin C, carotenoids, minerals, flavonoids, and phenolics, and we get them mainly from plants (fruits, vegetables, spices, beverages, nuts, and grain products) and animals (meats and fish) (Mirończuk-Chodakowska et al. 2018; Panova and Tatikolov 2023). Furthermore, exogenous antioxidative metabolites can also be obtained from other organisms, such as micro- and macroalgae, lichens, fungi, bacteria, and Archaea (Chandra et al. 2020; Wu et al. 2022; Sánchez-León et al. 2023).

## Diversity of bacteria

According to the molecular-based approach proposed by Woese et al. (1990), life forms on Earth are divided into three main domains: *Archaea*, *Bacteria*, and *Eukarya*. The domain *Eukarya* includes organisms that possess a well-defined nucleus enclosed within a two-layered membrane and other organelles within a distinct membrane, while the domains *Bacteria* and *Archaea* include prokaryotic organisms that lack a specific membrane-bound genome (nucleus) and organelles within their cytoplasm (Krishnan 2021; Lee et al. 2023; Lee and Ghos 2023).

The domain *Bacteria* encompasses numerous phyla; however, some phyla, such as *Actinobacteria*, *Bacteroidetes* (*Bacteroidota*), *Cyanobacteria*, *Firmicutes*, and *Proteobacteria* (*Pseudomonadota*), are well-known due to their industrial, biotechnological, and medicinal potentials, which have been widely examined. *Actinobacteria*, one of the largest phyla within the domain Bacteria, comprises spore-forming bacteria with a high GC content in their genomes. The members of this phylum are ubiquitously distributed in both aquatic (including marine) and terrestrial ecosystems. *Actinobacteria* can be either heterotrophic or chemoautotrophic, but most are chemoheterotrophic and utilize a wide variety of nutritional sources and complex polymers. The morphology of *Actinobacteria* members may vary depending on growth conditions; however, the majority of them display a typical mycelial organization similar to that of filamentous fungi. Although the majority of *Actinobacteria* members are aerobic, some genera within the phylum include facultative or obligate anaerobic species. *Actinobacteria* members are typically Gram-stain-positive or Gram-stain-variable (Barka et al. 2016; Li et al. 2016; Amin et al. 2020). According to “*Bergey’s Manual of Systematics of Archaea and Bacteria*,” the phylum *Actinobacteria* comprises five classes, 19 orders, 50 families, and 221 genera (Amin et al. 2020).

*Proteobacteria* are the largest and most phenotypically diverse phylogenetic phylum within the domain *Bacteria*. This phylum is divided into six classes, previously regarded as subclasses of the phylum (Rizzatti et al. 2017). *Proteobacteria* members are gram-negative and have a lipopolysaccharide layer in their outer membrane. The phylum also exhibits extreme diversity in energy-generating properties, as its members can be chemoorganotrophic, chemolithotrophic, or phototrophic. Regarding their relationship to oxygen, the phylum comprises strict aerobes, anaerobes, facultative aerobes, and microaerophiles (Kersters et al. 2006; Rizzatti et al. 2017).

*Cyanobacteria*, also known as blue-green algae or prokaryotic microalgae, comprise members that exhibit Gram-negative staining properties and perform oxygenic photosynthesis. They are distributed in water, terrestrial environments, and symbioses (Zutshi and Fatma 2015; Esteves et al. 2020). This phylum comprises approximately 2000 species that exhibit differences in terms of cell types, cellular structures, and physiological strategies (Esim et al. 2024).

Members of the phylum *Bacteroidetes* are distributed in various habitats, including soil, ocean, freshwater, and the gastrointestinal tracts of animals. The phylum consists of six bacterial classes that exhibit Gram-negative staining, are non-spore-forming, and are chemo-organotrophic. The members of the class *Bacteroidia* mostly colonize the gastrointestinal tracts, while members of other courses, including *Chitinophagia*, *Cytophagia*, *Flavobacteriia*, *Saprospiria*, and *Sphingobacteriia*, are mainly found in the natural environment (Thomas et al. 2011; Hahnke et al. 2016; Brinkmann et al. 2022).

According to the current classification, the members of the phylum *Firmicutes* are categorized into six classes: namely *Bacilli, Clostridia, Erysipelotrichia, Limnochordia, Negativicutes,* and *Tissierellia*. Most of them have a gram-positive cell wall structure and can produce endospores, which are resistant to desiccation and extreme environmental conditions. Spore-forming genera are distributed in most aquatic and terrestrial habitats (Galperin et al. 2022). Moreover, specific genera, such as *Enterococcus* and *Lactobacillus*, of the *Firmicutes* phylum colonize the gastrointestinal tract and are considered beneficial to humans (Power et al. 2014; Galperin et al. 2022).

## Production, extraction, purification, characterizatıon, and biological activities of bacterial metabolites

Bioactive metabolites of bacteria and fungi are produced in solid-state or submerged cultures. In solid-state cultures, moistened solid substrates that mimic a suitable environment for microbial growth are utilized. The amount of moisture in the substrate should be at a level that supports the development and metabolism of the selected microorganism. On an industrial scale, solid-state fermentations are conducted in trays, packed-bed reactors, horizontal drums, and fluidized-bed reactors (Prabhu et al. 2022). Submerged cultures are performed in shake flasks or bioreactors. Shake-flask cultures can be used for metabolite production; however, they are primarily used to screen the metabolite-producing abilities of microorganisms and to optimize culture conditions. At the same time, bioreactors are used for the large-scale production of metabolites (Dey et al. 2016; Srivastava et al. 2019; Takahashi et al. 2020). To increase the output of the targeted metabolite, the culture parameters (temperature, pH, incubation time, carbon and nitrogen sources, minerals, oxygen and carbon dioxide concentration, light density, shaking speed, and humidity rate) are optimized according to the requirement of the selected microorganism (Thomas et al. 2013; Dey et al. 2016; Singh et al. 2017a; Prabhu et al. 2022).

In the production of natural metabolites, using bacteria offers several advantages over plants and other organisms, including easy culturing in the laboratory, regardless of seasonal changes; rapid growth rates on simple and inexpensive media; easy manipulation of growth conditions; a low risk of contamination; and ease of modification through genetic engineering techniques. Besides, bacteria synthesize metabolites mostly extracellularly; therefore, the extraction of their metabolites is easier (Venil et al. 2013; Pinu and Villas-Boas 2017; López et al. 2023).

Extraction is the first step in methods used to separate bioactive metabolites from bacterial cells or their fermentation cultures. Some bioactive metabolites accumulate inside bacteria. Therefore, they are extracted from cell biomass using organic solvents. Moreover, to increase the effectiveness of organic solvents, cells are also treated with cell disruption methods simultaneously or before solvent application (Zainuddin et al. 2021; López et al. 2023). Cell disruption methods can be categorized into two main groups: mechanical methods and non-mechanical methods. The first group, namely mechanical methods, includes liquid (ultrasonication, microwaving, high-pressure homogenization) and solid shear methods (bead mill, grinding, and freeze press). The latter group comprises physical (osmotic shock and desiccation), chemical (acid, base, solvent, and detergent), and biological (enzyme and autolysis) treatment processes (Zainuddin et al. 2021). For example, most carotenoids are extracted from bacterial cells (cell biomass) using organic solvents alone, such as methanol, acetone, dimethyl sulfoxide, or ethyl acetate, or a combination of organic solvents with cell disruption methods (Nemer et al. 2021; Jiang et al. 2023; Rajendran et al. 2023). Similarly, phycobiliproteins and mycosporin-like amino acids are extracted from bacterial cells rather than fermentation broth (Sonani et al. 2017; Patel et al. 2018; Kokabi et al. 2022). On the contrary, bacterial metabolites such as polysaccharides, peptides, phenolics, and alkaloids are mainly secreted into the fermentation broth, so they are extracted directly from the fermentation broth using organic solvents after the bacterial cells are removed by centrifugation (Saravana Kumar et al. 2014; Tan et al. 2017, 2019; Dholakiya et al. 2017; Lee et al. 2020a; Xiong et al. 2020; Seo et al. 2022). For instance, for the isolation of extracellular bacterial polysaccharides, the fermentation broth is first centrifuged to remove bacterial cells and the polysaccharides in the resulting liquid fraction (supernatant) are then precipitated by cold ethanol treatment (Tiwari et al. 2019; Wang et al. 2019). Unlike plants, bacteria mainly secrete their metabolites outside the cell, that is, into the fermentation medium. Therefore, the extraction of the bacterial metabolites is easier.

**Fig. 2:**
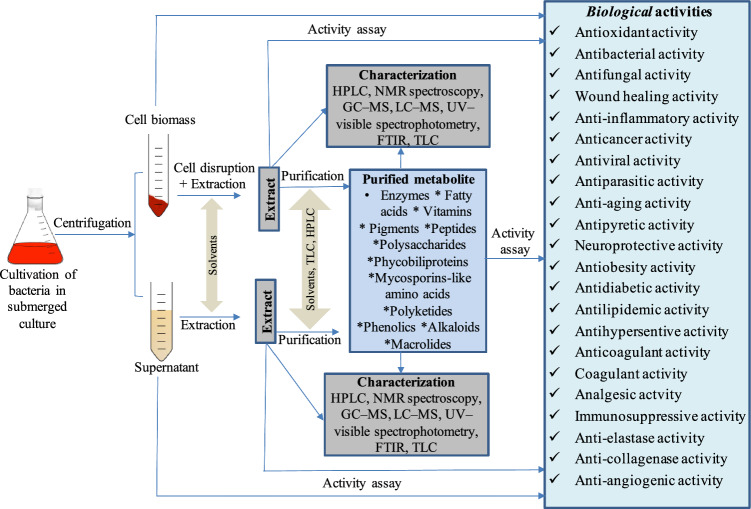
Production, extraction, purification, characterization, and biological activities of bacterial metabolites. Some bioactive metabolites are extracted from bacterial cells, while others are extracted from culture supernatants of bacteria. The chemical characterization of the purified metabolites or the extracts containing these metabolites was performed using the apparatus such as high-performance liquid chromatograph (HPLC), ultraviolet–visible spectrophotometry (UV–VIS), fourier transform infrared spectroscopy (FTIR), gas chromatography–mass spectrometry (GC–MS), and liquid chromatography–mass spectrometry (LC–MS). The culture supernatants, extracts, and purified metabolites of bacteria exhibit diverse bioactive properties, including antioxidant, anticancer, antibacterial, antiviral, and wound-healing activities

It is known that even if metabolites are extracted from bacterial cells or their fermentation broth, they may still contain undesirable impurities such as proteins and lipids. Accordingly, these impurities must be removed from the target metabolite using appropriate purification methods. For instance, purification methods such as column chromatography, preparative thin-layer chromatography (TLC), and high-performance liquid chromatography (HPLC) are employed to separate the extracted pigments from impurities, including lipids and proteins (Yu et al. 2022; Rajendran et al. 2023). Later, the purified bacterial pigments are structurally identified using Raman spectroscopy, HPLC, ultraviolet–visible spectrophotometry (UV–VIS), fourier transform infrared spectroscopy (FTIR), energy-dispersive X-ray spectroscopy (EDX), nuclear magnetic resonance (NMR) spectroscopy, gas chromatography–mass spectrometry (GC–MS), and liquid chromatography–mass spectrometry (LC–MS) (Jinendiran et al. 2020; Soni et al. 2023; Rajendran et al. 2023) (Fig. 2).

Bacteria-derived natural products are mainly tested in their extract form or pure form. Their extracts and/or purified metabolites exhibit various biological activities, including antioxidant, antibacterial, antifungal, wound healing, anti-inflammatory, anticancer, antiviral, antiparasitic, anti-aging, neuroprotective, antiobesity, antidiabetic, antilipidemic, antihypersentive, anti-collagenase, anti-angiogenic, anticoagulant, coagulant, antipyretic and analgesic activities (Petrosyan et al. 2012; Ayed et al. 2015; Jung et al. 2015; Hassan 2016; Zouari et al. 2016; Sonani et al. 2017; Arivizhivendhan et al. 2018; Shirzad et al. 2018; Lozano-González et al. 2019; Khushboo et al. 2022; Bharathi et al. 2023; Abdelaziz et al. 2024; Esim et al. 2024). Additionally, the direct culture supernatants (cell-free filtrates) of bacteria exhibit antimicrobial, anticancer, and antioxidant properties (Rajoka et al. 2019; Yang et al. 2019; Abd Ellatif et al. 2022). Lactic acid bacteria can be directly added to creams, gels, or wound dressing materials to enhance wound healing (Sinha et al. 2019; Brandi et al. 2020). They are also used as probiotics in the prevention or alleviation of various health disorders, including obesity, diabetes, infections, cancer, neurodegenerative diseases, and inflammatory bowel disease (Hussain et al. 2020; Olvera-Rosales et al. 2021; Dasdemir et al. 2024). In short, the cells, culture supernatants, extracts, and pure metabolites of bacteria exhibit various biological activities (Fig. 2).

## Bacteria-derived antioxidant compounds: types and activities

**Fig. 3:**
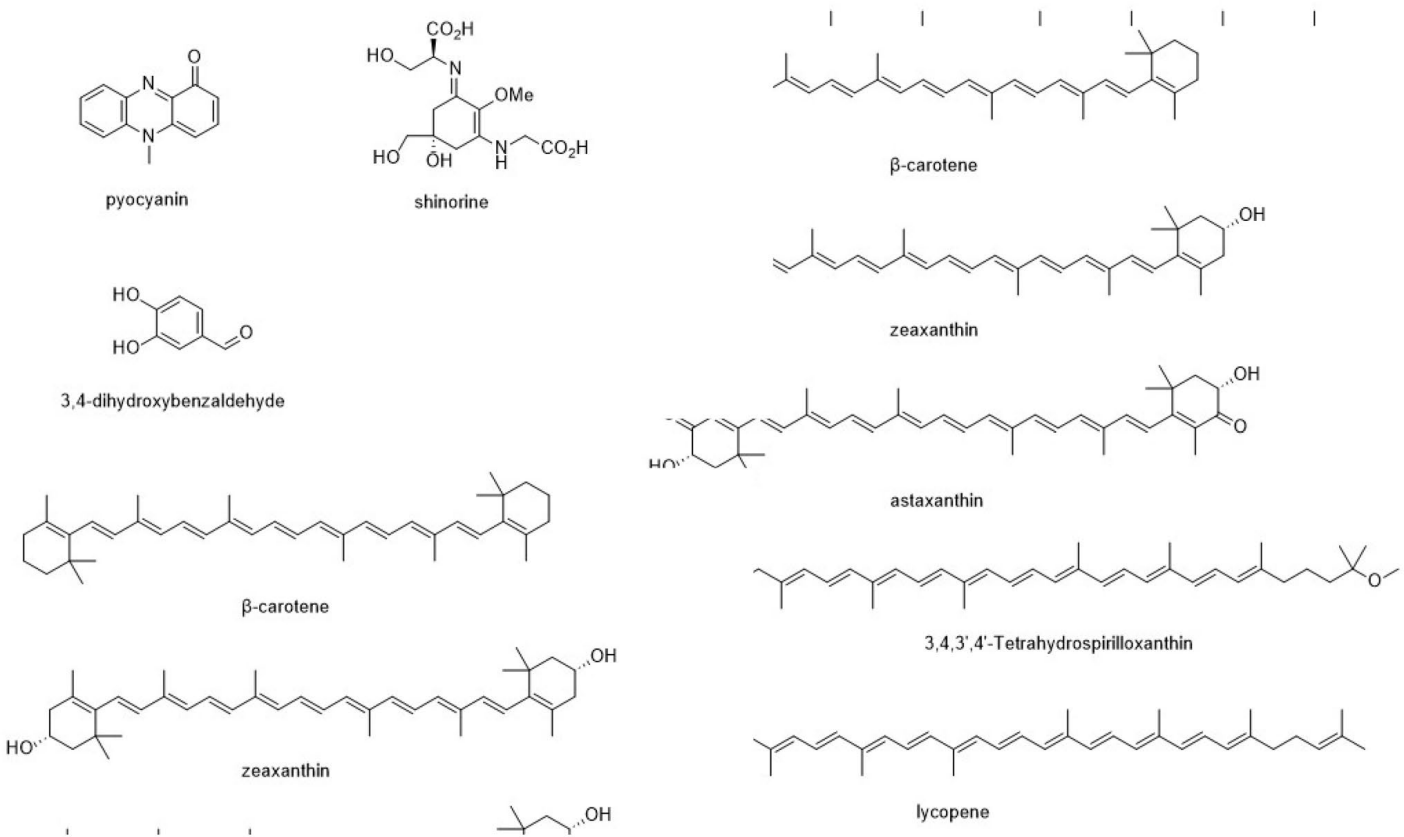
Chemical structures of bacteria-derived metabolites with antioxidant activity

There are several pathogenic bacterial species, including the members of the *Proteobacteria, Bacteroidetes, Firmicutes*, and *Actinobacteria* (Wexler 2007; Thomas et al. 2011; Bien et al. 2013; Rizzatti et al. 2017; Wu et al. 2021); however, the majority of bacteria are considered beneficial to humans and therefore are widely used for various purposes in health, veterinary, agriculture, pharmaceutical, food, cosmetic, detergent, and textile industries (Vitorino and Bessa 2017; Douillard et al. 2019). For instance, the culture supernatants and/or cell biomass of several members of the phyla *Actinobacteria, Proteobacteria, Bacteroidetes, Firmicutes*, and *Cyanobacteria* can be used for the purification of different metabolites with antioxidant activity, such as pigments, polysaccharides, peptides, phycobiliproteins, mycosporins-like amino acids, phenolics, alkaloids, and benzoic acid esters. The chemical structures of some metabolites with antioxidant activity from bacteria are shown in Fig. 3.

**Fig. 4:**
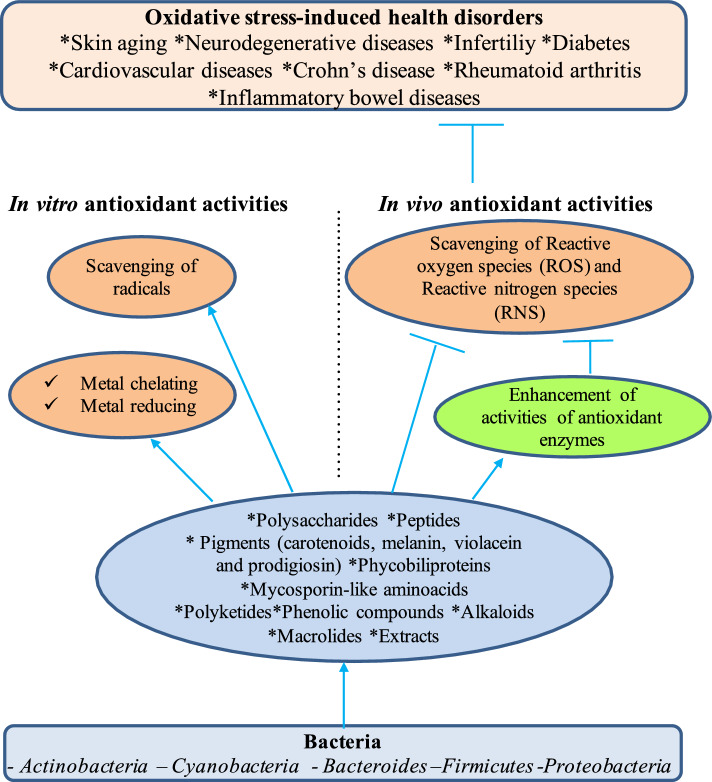
In vitro and in vivo antioxidant activities of bacteria-derived metabolites and extracts. Species belonging to different bacterial phyla, including *Actinobacteria*, *Proteobacteria*, *Bacteroidetes*, *Firmicutes*, and *Cyanobacteria*, produce various metabolites with antioxidant activity, such as pigments, polysaccharides, peptides, phycobiliproteins, mycosporine-like amino acids, phenolic compounds, alkaloids, and benzoic acid esters. The purified metabolites or extracts containing these metabolites exhibit in vitro antioxidant activities in radical scavenging, metal-reducing, and metal-chelating assays. They show in vivo antioxidant activities by scavenging directly reactive oxygen species (ROS) and reactive nitrogen species (RNS), and/or by indirectly eliminating ROS and RNS via the enhancement of antioxidant enzyme activities

The extracts and/or purified metabolites exhibit in vitro antioxidant potency in radical scavenging, metal-reducing, and metal-chelating assays. Their in vitro radical scavenging activities are mainly tested against 2,2′-azino-bis(3-ethylbenzothiazoline-6-sulfonic acid (ABTS), 2,2-Diphenyl-1-picrylhydrazyl (DPPH), H_2_O_2_, ·OH, O_2_·^−^ and NO^·^ radicals. They display the in vivo antioxidant activities by scavenging directly ROS/RNS (·OH, O_2_·^−^, H_2_O_2_, NO^·^) and/or by eliminating indirectly ROS/RNS via the enhancement of activities of antioxidant enzymes (SOD, CAT and GPx) (Fig. 4) (Manivasagan et al. 2013; Saravana Kumar et al. 2014; Sajjad et al. 2018; Kemung et al. 2020; Sheefaa and Sivaperumal 2022; Djebbah et al. 2022).

### Pigments

Carotenoids exhibit a range of colors, including yellow, orange, red, and purple. Carotenoids can be produced by a diverse range of organisms, including bacteria, eukaryotic microalgae, macroalgae, plants, archaea, and fungi. They are classified into two main groups: carotenes (oxygen-free) and xanthophylls (oxygen-containing) (Arslan et al. 2023; Esim et al. 2024).

In humans, carotenoids primarily function as provitamin A, which helps protect the body against macular degenerative diseases. They also exhibit diverse bioactive properties, including antioxidant, anticancer, anti-inflammatory, and antimicrobial activities. Due to their numerous beneficial properties, they find applications in the pharmaceutical, cosmetic, and food industries. In the food industry, carotenoids are considered healthy food colorants (Montero-Lobato et al. 2018; Ávila-Román et al. 2021). There have been attempts to investigate the antioxidant potential of carotenoids derived from various bacterial species (Table 1). For instance, Jiang et al. (2023) investigated the antioxidant activity of the crude carotenoids from *Rhodococcus* aetherivorans N1, a member of *Actinobacteria*. They found that the crude carotenoid extract, containing β-carotene, zeaxanthin, and isorenieratene, displayed DPPH radical-scavenging activity. In a separate study (Yu et al. 2022), a novel carotenoid pigment was extracted from the actinobacterium *Arthrobacter* sp. QL17 was characterized and then tested for its potential biological activities. Based on structural analysis, the novel pigment was identified as arthroxanthin. This pigment exhibited strong antioxidant activity against DPPH and ABTS radicals, as well as moderate anticancer potency against various cell lines. A different research team (Metwally et al. 2022) focused on the chemical composition and bioactive properties of the carotenoid extract from the actinobacterium *Kocuria* sp. RAM1. The extract was found to contain three different red pigments: two C_50_-carotenoids (bisanhydrobacterioruberin and trisanhydrobacterioruberin) and one C42-carotenoid (3,4,3ʹ,4ʹ-Tetrahydrospirilloxanthin). The carotenoid extract displayed antioxidant potential in the DPPH assay. Furthermore, the extract has been demonstrated to possess antimicrobial, anti-inflammatory, antiviral (against HSV-1), anticancer, antidiabetic, and wound-healing properties. Jaber and et al. (2021) demonstrated that β-carotene from the proteobacterium Paracoccus homiensis strain BKA7 possessed strong antioxidant potential in scavenging the DPPH radical. They also reported that β-carotene exhibited high antimicrobial activity against both Gram-positive and Gram-negative bacteria. In a recent study (Naik and Gupte 2024), the pigments of *Paracoccus marcusii* RSPO were extracted, purified, characterized, and subsequently evaluated for their potential antioxidant and antimicrobial properties. The carotenogenic pigment extract was found to contain astaxanthin, zeaxanthin, ζ-carotene, and β-zeacarotene. The extract exhibited inhibitory effects against α-amylase and α-glucosidase, and showed an antibacterial effect against Escherichia coli and *Enterobacter aerogenes.* Moreover, the results from the in vitro antioxidant activity assays displayed that the carotenoid extract had the potential to scavenge DPPH and ABTS radicals. A recent study by Soni and co-workers (2023) focused on the partial characterization and antioxidant activity of an orange pigment extracted from the marine bacterium, a member of the phylum *Firmicutes*. The extracted pigment was identified as a carotenoid pigment group. The pigment was found to have antibacterial efficacy against both Gram-negative and Gram-positive bacteria. The in vitro antioxidant activity assays revealed that the pigment possessed the ability to scavenge ABTS, DPPH, and H_2_O_2_ radicals, and also exhibited ferric reducing power activity. A different study (Gurkok 2022) clarified that the carotenoid pigment with an orange color from the *Metabacillus idriensis* strain LipT27 exhibited in vitro antioxidant potential against DPPH and ABTS radicals, as well as antibacterial effectiveness. Sricharoen et al. (2022) investigated the antioxidant activities of crude pigment extracts from five bacterial species (*Halobacillus yeomjeoni, Salinicoccus sp., Bacillus infantis, B. Amyloliquefaciens,* and *Staphylococcus carnosus*) from the phylum *Firmicutes*. The structural analyses uncovered that the crude pigment extracts contained carotenoid derivatives. Based on the results of the DPPH assay, the crude carotenoid extract of *B. amyloliquefaciens* was ascertained to have higher antioxidant activity compared to those of other bacteria. Jinendiran et al. (2020) investigated the biological activities, including anti-cancer, anti-inflammatory, and antioxidant potentials, of carotenoids from the methanolic extract of *Exiguobacterium acetylicum* S01. The methanolic extract was determined to contain six different carotenoids: lycopene (Car-I), diapolycopenedioic acid diglucosyl ester (Car-II), β-carotene (Car-III), zeaxanthin (Car-IV), astaxanthin (Car-V), and keto-myxocoxanthin glucoside ester (Car-VI). All carotenoids exerted a notable cytotoxic effect on HT-29 cells and also inhibited lipopolysaccharide-induced nitric oxide production, tumor necrosis factor-alpha (TNF-α), and lipid peroxidation in peripheral blood mononuclear cells. Furthermore, the DPPH radical-scavenging assay revealed that Car-II and Car-VI exhibited higher antioxidant activity than the standard control, ascorbic acid. In a study by Fariq et al. (2019), the pigments of three halophilic bacteria, *Aquisalibacillus elongatus* MB592 and *Salinicoccus sesuvii* MB597 from the phylum *Firmicutes*, and *Halomonas aquamarina* MB598 from the phylum *Proteobacteria*, were structurally analyzed and subsequently tested for their potential antioxidant activities. Analytical techniques elucidated that the purified pigments were the derivatives of bacterioruberin carotenoids. The in vitro assays revealed that the purified pigments possessed antioxidant (DPPH radical-scavenging) and antimicrobial activities, including antifungal and antibacterial properties. Silva et al. (2019) studied the antioxidant potential of crude pigment extracts from Antarctic bacteria. They reported that the C50 carotenes (bacterioruberin and decaprenoxanthin) were present in the pigment extracts of *Actinobacteria* strains *(Arthrobacter agilis* 50cyt and *A. psychrochitiniphilus* 366). In contrast, the pigment extract of *Zobellia laminarie* 465 *(Bacteriodes)* contained Zeaxanthin, β-cryptoxanthin, β-carotene, and phytoene. They found that the crude pigment extracts from these strains exhibited the in vitro antioxidant activities in DPPH and ABTS radicals-scavenging and ORAC assays.

**Table 1:** Antioxidant and other biological activities of bacteria-derived pigments

Compound	Bacterium–Phylum	Activity	References
Carotenoid extracts (β-carotene, zeaxanthin and isorenieratene)	*Rhodococcus aetherivorans* N1 (*Actinobacteria*)	DPPH radical-scavenging (at the concentration of 25 mg/L, the scavenging capacity of crude carotenoids was more than 80%, while that of pure *β*-carotene was only 65%) (In vitro)	(Jiang et al. 2023)
Arthroxanthin	*Arthrobacter *sp. QL17 (*Actinobacteria*)	DPPH and ABTS radicals-scavenging activities (IC50s of 69.8 and 21.5 µg/mL, respectively) as well as anticancer potency against HepG2, Hela, MDAB-231, SW480, and MKN-45 cell lines (In vitro)	(Yu et al. 2022)
Bisanhydrobacterioruberin, Trisanhydrobacterioruberin and 3,4,3ʹ,4ʹ-tetrahydrospirilloxanthin (carotenoids)	*Kocuria* sp. RAM1 (*Actinobacteria*)	DPPH radical-scavenging (67.99% at the highest concentration of 1000 µg/mL, IC50 = 59.67 µg/mL), antimicrobial (against *S. aureus, B. subtilis, E. faecalis, E. coli*, *K. pneumoniae,* and *P. aeruginosa*), anti-inflammatory, anti-HSV-1, anticancer (against MCF-7, Caco-2 and HeLa), antidiabetic (α-glucosidase inhibition), and wound healing activities (In vitro)	(Metwally et al. 2022)
β-carotene	*Paracoccus homiensis* BKA7 (*Actinobacteria*)	DPPH radical-scavenging activity and antibacterial effectiveness (against *S. aureus, E. coli*, *B. cereus,* and *P. aeruginosa*) (In vitro)	(Jaber et al. 2021)
Astaxanthin, zeaxanthin, ζ-carotene and β-zeacarotene (carotenoids)	*Paracoccus marcusii* RSPO1 (*Actinobacteria*)	DPPH and ABTS radicals-scavenging activities (65% and 42% respectively at the concentration of 20 µg mL) as well as antibacterial (against *E. coli* and *E. aerogene*s) and anti-diabetic (α-amylase and α-glucosidase) activities (In vitro)	(Naik and Gupte 2024)
Carotenoids	*Bacillus infantis (Firmicutes)*	Scavenging activities towards DPPH, ABTS and H_2_O_2_ (71%, 83.4% and 60.75% at a concentration of 500 µg/mL, respecitvely). Also, antibacterial efficiency against *P. aeruginosa, S. dysenteriae, S. enterica ser. typhi, S. marcescens, B. megaterium, S. aureus* and *S. epidermis* (In vitro)	(Soni et al. 2023)
An orange pigment (carotenoid)	*Metabacillus idriensis* LipT27 *Firmicutes)*	Radical-scavenging activity (IC_50_ values of 72 µg/mL and 26 µg/mL for ABTS and DPPH, respectively) and antibacterial effects (against *Yersinia enterocolitica*, *Staphylococcus aureus*, and *Escherichia coli)* (In vitro)	(Gurkok 2022)
Carotenoids	*Bacillus amyloliquefaciens (Firmicutes)*	DPPH radical scavenging activity (14.93% and 95.5% for carotenoid extract and AA, respectively) (In vitro)	(Sricharoen et al. 2022)
Different fractions of carotenoids (Lycopene, diapolycopenedioic-acid-diglucosyl-ester, β-carotene, zeaxanthin, astaxanthin)	*Exiguobacterium acetylicum* S01 *(Firmicutes)*	DPPH scavenging activities (IC50 values of the fractions Car-II and Car-VI were less than the value of AA, while those of the fractions Car-I and Car-IV were significantly higher than AA). Reduction of NO^·^ and lipid peroxidation levels in s in LPS-induced peripheral blood mononuclear cells. Besides, anticancer effect against HT-29 cell line (In vitro)	(Jinendiran et al. 2020)
Bacterioruberin (carotenoids)	*Aquisalibacillus elongatus* MB592 and *Salinicoccus sesuvii* MB597 *(Firmicutes), Halomonas aquamarina* MB598 (*Proteobacteria*)	DPPH scavenging activity (85%) as well as antibacterial (against *B. subtilis, B. pumilus, B. cereus, E. faecalis, E. faecium, K. pneumoniae, A. faecalis* and *P. geniculata*) and antifungal (against A*. fumigatus, A. flavus, F. solani,* and *Mucor* spp.) activities (In vitro)	(Fariq et al. 2019)
The extracts containing bacterioruberin, decaprenoxanthin, Zeaxanthin, β-cryptoxanthin, β-carotene, and phytoene	*Arthrobacter agilis* 50cyt and *A. psychrochitiniphilus* 366 (*Actinobacteria*) and *Zobellia laminarie* 465 (*Bacteriodes)*	ABTS (5.31, 3.38 and 4.37 μmol Trolox equivalent TE/mg of pigment for *A. agilis, A. psychrochitiniphilus* and *Zobellia laminarie,* respectively) and DPPH radicals-scavenging activities (5.95, 3.24 and 4.59 μmol Trolox equivalent TE/mg of pigment for *A. agilis, A. psychrochitiniphilus* and *Zobellia laminarie,* respectively) (In vitro)	(Silva et al. 2019)
Prodigiosin	*Streptomyces* sp. strain WMA-LM31(*Actinobacteria*)	DPPH radical scavenging (62.51% and 69.2% for the prodigiosin and AA at the concentration of 10.0 µg/mL), protein oxidation inhibition (54.82% and 43.2% for the prodigiosin and AA), lipid peroxidation inhibition (25.4% and 42.48% for the prodigiosin and AA) activities, as well as anticancer effectiveness against HeLa and HepG2 cell lines (In vitro)	(Sajjad et al. 2018)
The extract containing spectinabilin (a polyketide metabolite) and two prodigiosins (undecylprodigiosin and metacycloprodigiosin)	*Streptomyces* V002 (*Actinobacteria*)	ABTS (IC_50_ values of 5.8 10^–3^ and 0.98 10^–3^ µg/µL for the extract and trolox) and DPPH (IC_50_ values of 0.834 and 0.25 10^–3^ µg/µL for the extract and trolox) scavenging activities and antibacterial effectiveness (*C. violaceum, S. aureus, Micrococcus luteus, Mycobacterium smegmatis, B. subtilis* (In vitro)	(Gacem et al. 2020)
Prodigiosin	*Serratia marcescens* CSK (*Proteobacteria*)	DPPH and ABTS radicals-scavenging activities (78% and 71% at the concentration of 500 μg/mL, respectively) as well as antimicrobial and anticancer effectiveness (In vitro)	(Sudhakar et al. 2022)
Prodigiosin	*S. marcescens* MB703 (*Proteobacteria*)	Antioxidant (the increases in SOD and CAT but the decrease in MDA level in normal SH-SY5Y human neuroblastoma cells) and anticancer (against HT-29 human colon cancer and SK-MEL-30 human melanoma cells) activities (In vitro)	(Koyun et al. 2022)
Prodigiosin	*Janthinobacterium* ERMR3 (*Proteobacteria*)	ABTS and DPPH radicals-scavenging activities (IC50 values of 13.3 μg/mL for DPPH and 11.39 μg/mL for ABTS, respectively) (In vitro)	(Mukhia et al. 2023)
Melanin	*Streptomyces glaucescens* NEAE-H (*Actinobacteria*)	ABTS radical scavenging (57.2% and 89.6% for the melanin and AA at the concentration 100 μg/mL) and anticancer (against HFB4 skin cancer cell line) activities (In vitro)	(El-Naggar et al. 2017)
Melanin	*Streptomyces* spp. (*Actinobacteria*)	DPPH scavenging (93.47% and 98.6% respectively for the melanin and AA at the concentration 150 μg/mL) and lipid peroxidation inhibition (48.62% and 72.62% respectively for the melanin and AA at the concentration 100 μg/mL) activities (In vitro)	(Sheefaa and Sivaperumal 2022)
Pyomelanin	*Streptomyces djakartensis* NSS-3 (*Actinobacteria*)	DPPH scavenging (IC_50_ values of 18.03 µg/mL and 16.83 µg/mL for the melanin and AA), photoprotective, anticancer (against HCT116, HEPG, and MCF7 cell lines) and antibacterial (against *S. aureus, E. coli, K. pneumoniae,* and *P. aeruginosa)* activities (In vitro)	(El-Zawawy et al. 2024)
Eumelanin	*Providencia rettgeri* strain BTKKS1 (*Proteobacteria*)	DPPH radical-scavenging (63.73%) and ferric-reducing power (97.09%) activities as well as anti-inflammatory activity (inhibition of cyclooxygenase, lipoxygenase, myeloperoxidase, and cellular nitrite in a dose-dependent manner) (In vitro)	(Kurian and Bhat 2017)
Melanin	*Rhizobium radiobacter* ATCC 1333 (*Proteobacteria*)	·OH (84.12%) and DPPH (89.74%) radicals-scavenging activites as well as antibacterial effectiveness against *E. coli*, *S. aureus*, *A. baumanii*, and *M. albican* (In vitro)	(Wu et al. 2022)
Melanin	*Pseudomonas mosselii* STSGRDS1 (*Proteobacteria*)	·OH (84.75%), NO^·^ (55.47%), DPPH (66.96%), ABTS (26.13%) and H_2_O_2_ (25.8%) radicals-scavenging and ferrric reducing power activities (In vitro)	(Mary et al. 2020)
Melanin	*Gluconobacter oxydans* FBFS 97 (*Proteobacteria*)	DPPH and ABTS radicals-scavenging activities (IC_50_ values of 36.94 µg/mL and 14.06%, respectively) (In vitro)	(Noman et al. 2022)
Eumelanin	*Bacillus subtilis* 4NP-BL *(Firmicutes)*	DPPH radical-scavenging activity (94.47% at a concentration of 100 μg/mL, and the IC50 values of 37.17 and 38.93 μg/mL for the melanin and AA, respectively) and antibacterial effectiveness against *Xanthomonas campestris* and *Alteromonas macleodii* (In vitro)	(Ghadge et al. 2020)
Violacein	*Chromobacterium violaceum* (*Proteobacteria*)	DPPH and ABTS radicals-scavenging activities (IC_50_ values of 0.286 and 0.182 g/L, respectively) and antibacterial effecttiveness against *S. aureus* and *B. subtilis* (In vitro)	(Cheng et al. 2022)
Violacein	*Chromobacterium vaccinii *CV5 (*Proteobacteria*)	DPPH, O_2_^·–^, NO^·^, H_2_O_2_ and ·OH radicals scavenging activities (IC_50_ values of 297.88 µg/mL, 312.89 µg/mL, 410.17 µg/mL, 296.74 µg/mL and 292.74 µg/mL, respectively) (In vitro)	(Vishnu and Palaniswamy 2018)
Pyocyanin	*Pseudomonas aeruginosa* (*Proteobacteria*)	DPPH (58%, 68.5% and 88.1% for pyocyanin, Trolox and BHT, respectively) and ABTS (52.5%, 67.4% and 86.0% for pyocyanin, Trolox and BHT, respectively) radicals-scavenging activities as well as antibacterial, antifungal and anti-biofilm activities (In vitro)	(Saleem et al. 2021)
4,8,13-trihydroxy-6,11-dione-trihydrogranaticins (a blue pigment)	*Streptomyces* sp*.* A1013Y (*Actinobacteria*)	DPPH and ABTS radicals scavenging activities (IC_50_ values of 41.04 µg/mL and 13.75 µg/mL respectively) (In vitro)	(Zhu et al. 2020)
Yellow-orange color pigment	*Streptomyces* sp. D25 (*Actinobacteria*)	DPPH (35.63%) and NO^·^ (96.19%) radicals- scavenging activities as well as antimicrobial effectiveness against biofilm-forming strains of *Staphylococcus Lactobacillus, Alcaligens Bacillus* and *Micrococcuss* (In vitro)	(Radhakrishnan et al. 2016)
Yellow-colored pigments	*S. maritimus* AJ6 and *S. fenghuangensis* AJ7	Antioxidant (the scavenging values of 39%, 41% and 72% respectively for AJ6, AJ7 and AA against DPPH at a pigment concentrationof 500 µg/mL, antimicrobial, anti-inflammatory, antiproteinase, and antifouling activities (In vitro)	(Nair and Abraham 2022)

*ABTS*, 2,2'-azino-bis(3-ethylbenzothiazoline-6-sulfonic acid, *DPPH* 2,2-Diphenyl-1-picrylhydrazyl, ·*OH* hydroxyl radical, *O*_*2*_^·–^ superoxide radical, *H*_*2*_*O*_*2*_ hydrogen peroxide, *NO*^·^ nitric oxide radical, *SOD* superoxide dismutase, *CAT* catalase and *GPx* glutathione peroxidase, *BHT* Butylated hydroxytoluene, *AA* ascorbic acid and Trolox represent the control compounds used in antioxidant activity assays

Prodigiosin is a light-sensitive, red pigment produced by bacteria. The pigment is water insoluble but soluble in chloroform, methanol, acetonitrile, and DMSO. Additionally, it has moderate solubility in alcohol and ether (de Araújo et al. 2010). The pigment exhibits various biological activities, including antioxidant, antibacterial, antifungal, antimalarial, anticancer, antidiabetic, and immunosuppressive properties (Arivizhivendhan et al. 2018; Bharathi et al. 2023). In the phylum *Actinobacteria*, members of the genus *Streptomyces* are potential producers of prodigiosin (Sajjad et al. 2018; Gacem et al. 2020). The prodigiosin-producing species in the phylum *Proteobacteria* include Serratia marcescens, *S. rubidaea*, *Pseudomonas magneslorubra*, *Vibrio psychroerythrous*, *V. gazogenes*, and *Alteromonas rubra* (Darshan and Manonmani 2015; Mukhia et al. 2023). Several studies have investigated the antioxidant potential of prodigiosin from these bacterial strains (Table 1). For example, Sajjad and coworkers (2018) reported that prodigiosin from the radioresistant *Streptomyces* sp. strain WMA-LM31 could scavenge DPPH radicals and inhibit protein and lipid peroxidation. Furthermore, the results of their study provide evidence that prodigiosin has an anticancer effect on HeLa and HepG2 cell lines in a dose-dependent manner. A work conducted by Gacem and et al. (2020) revealed that the ethyl acetate extract of the isolate V002 (*Streptomyces* strain) contained spectinabilin (a rare polyketide metabolite) and two prodigiosin forms (undecylprodigiosin and metacycloprodigiosin), and that the extract had the antioxidant potential in DPPH and ABTS radicals-scavenging assays and also exhibited antibacterial effectiveness towards different pathogens. Sudhakar and co-workers (2022) reported that the prodigiosin from *S. marcescens* strain CSK exhibited vigorous antioxidant activity in scavenging DPPH and ABTS radicals in a dose-dependent manner. Furthermore, they determined that prodigiosin possesses antimicrobial and anticancer properties. A different research group (Koyun et al. 2022) reported that prodigiosin from *S. marcescens* MB703 exhibited anticancer activity against HT-29 human colon cancer and SK-MEL-30 human melanoma cells, without causing genotoxic or cytotoxic effects in L929 healthy mouse fibroblasts. Furthermore, they found that the prodigiosin can protect SH-SY5Y human neuroblastoma cells against oxidative stress by increasing antioxidative enzyme activities (SOD and CAT) and decreasing MDA level. In a separate study conducted by Mukhia and et al. (2023), the prodigiosin from the cold-adapted strain ERMR3 of the proteobacterium *Janthinobacterium* was shown to exhibit potent antioxidant activity in DPPH and ABTS radical-scavenging assays.

Melanin refers to a group of dark brown to black pigments synthesized by animals, fungi, and bacteria. There are five main categories of melanin, namely, eumelanin, pheomelanin, neuromelanin, allomelanin, and pyomelanin. Melanins protect several fungal and bacterial species from UV, solar, and gamma radiation, as well as heavy metal toxicity, and also increase their chances of survival in extreme conditions. Furthermore, melanins enable bacteria to interact with other organisms and increase their virulence (Nosanchuk and Casadevall 2006; Pavan et al. 2020; Singh et al. 2021; Ghattavi et al. 2022). In addition to their beneficial roles in bacteria, the melanins derived from bacteria are also crucial to humans due to their potential bioactive properties, including antioxidant, anti-inflammatory, antimicrobial, photoprotective, and anticancer activities (El-Naggar et al. 2017; Kurian and Bhat 2017; Suwannarach et al. 2019). For example, the antioxidant potential of bacterial melanins has been evaluated in several earlier studies (Table 1). In a previous study, El-Naggar and coworkers (2017) reported that the melanin extracted from the actinobacterium *Streptomyces glaucescens* NEAE-H exhibited strong ABTS radical-scavenging potency, comparable to that of the standard antioxidant ascorbic acid. Furthermore, the melanin was found to possess notable cytotoxicity towards the HFB4 skin cancer cell line. In another study, Sheefaa and Sivaperumal (2022) found that the melanin from a marine *Streptomyces* species exhibited DPPH radical-scavenging and lipid peroxidation inhibition activities. In a recent study by El-Zawawy and et al. (2024), the pigment from *S. djakartensis* NSS-3 was characterized, and its biological activities were evaluated. Based on the data obtained from UV–VIS, FT-IR, EDX, and NMR analyses, the pigment was identified as a nitrogen-free pyomelanin. The results of the study also indicated that pyomelanin has strong DPPH radical-scavenging, radioprotective, anticancer, and antibacterial activities. Kurian and Bhat SG (2017) reported that the melanin from the marine proteobacterium *Providencia rettgeri* strain BTKKS1 exhibited radical-scavenging activity, metal-chelating potential, and anti-inflammatory properties. In a study conducted by Wu and et al. (2022), the physico-chemical properties and biological activities of the melanin from the proteobacterium *Rhizobium radiobacter* ATCC 1333 were investigated. The purified melanin was found to be soluble in various alkaline solutions but was hardly soluble in water and most organic solvents. The in vitro assays revealed that the melanin possessed antioxidative potency, capable of scavenging DPPH and ·OH radicals. Furthermore, melanin has been proven to have antimicrobial effectiveness against various pathogenic bacteria. Mary and et al. (2020) evaluated the in vitro antioxidant and photoprotective properties of melanin from the proteobacterium Pseudomonas mosselii strain STSGRDS1. They found that the melanin exhibited radical-scavenging activity against DPPH, ABTS, OH·, H_2_O_2_, and NO· radicals, as well as a ferric-reducing power property. The experiments also demonstrated that the melanin could protect against UV-A and UV-B rays. Due to its photoprotection potential and radical-scavenging activity, the melanin was recommended as an active ingredient in cosmetic formulations and UV protection devices. Noman et al. (2022) reported that the melanin from the proteobacterium *Gluconobacter oxydans* FBFS 97 exhibited significant scavenging activities against DPPH and ABTS radicals. Ghadge et al. (2020) investigated the biological activities of natural melanin from *Bacillus subtilis* 4NP-BL, an endophytic bacterium belonging to the phylum *Firmicutes*. The structural analyses clarified that the melanin belongs to the eumelanin class. Biological activity assays revealed that the melanin exhibited potent antioxidant activity against DPPH radicals, comparable to that of the standard ascorbic acid. Additionally, it was found that melanin exhibited antibacterial effects against *Xanthomonas campestris* and *Alteromonas macleodii*.

In addition to carotenoids, melanin, and prodigiosin, other pigments produced by bacteria have also been shown to possess antioxidant potential (Table 1). For example, in the phylum *Proteobacteria,* strains such as *Chromobacterium violaceum*, *C. vaccinii* CV5, *Janthinobacterium lividum*, *Duganella* sp., *Pseudoalteromonas* sp., *Iodobacter* sp., and *Massilia* sp. are capable of synthesizing violacein, a purple-colored pigment. It has been documented that violacein exhibits diverse bioactive properties, including antioxidant, antimicrobial, and anticancer effects (Masuelli et al. 2016; Vishnu and Palaniswamy 2018; Park et al. 2021; Cheng et al. 2022). For instance, Cheng et al. (2022) found that violacein from *Chromobacterium violaceum* exhibits potent antioxidant activity against DPPH and ABTS radicals, as well as strong antibacterial effectiveness against Gram-positive pathogens. Vishnu and Palaniswamy (2018) found that violacein from *C. vaccinii* CV5 exhibited potent antioxidant activity in DPPH, O_2_·^−^, ·OH, H_2_O_2_, and NO· radical scavenging assays. Similarly, Saleem and co-workers (2021) reported that the proteobacterium *Pseudomonas aeruginosa* can produce other pigments, including pyocyanin (blue-green), pyomelanin (light brown), pyoverdin (yellow, green, and fluorescent), and pyorubrin (red-brown). These researchers revealed that pyocyanin purified from *P. aeruginosa* exhibited antioxidant potential against DPPH and ABTS radicals, as well as antimicrobial effectiveness against foodborne pathogens (bacteria and fungi), and biofilm-forming bacteria.

The literature survey revealed that *Streptomyces* members also produce other pigments with antioxidant activity, in addition to carotenoids, melanin, and prodigiosin (Table 1). For example, Zhu and et al. (2020) investigated the physicochemical properties and antioxidant potency of a blue pigment extracted from the fermentation broth of *Streptomyces* sp*.* A1013Y. Based on the structural analyses, the pigment was identified as 4,8,13-trihydroxy-6,11-dione-trihydrogranaticins A. In vitro antioxidant activity assays elucidated that the blue pigment had an excellent scavenging capacity towards DPPH and ABTS radicals. In a separate study, Radhakrishnan et al. (2016) investigated the antioxidant and antimicrobial properties of the crude pigment, characterized by a yellow-orange color, from *Streptomyces* sp. D25, and they elucidated that the pigment exhibited antimicrobial activity against various biofilm-forming bacteria and also demonstrated antioxidant properties in NO· and DPPH radical-scavenging assays. Nair and Abraham (2022) found that the yellow-colored pigments extracted from *S. maritimus* AJ6 and *S. fenghuangensis* AJ7 could scavenge DPPH radical and also exhibit antimicrobial, anti-inflammatory, antiproteinase, and antifouling activities.

### Phycobiliproteins

Phycobiliproteins (PBPs), which are colored and water-soluble disk-shaped proteins, act as light-harvesting complexes in cyanobacteria and micro- and macroalgae from the phylum *Rhodophyta*. According to their light absorption characteristics, PBPs are classified into four distinct groups: phycoerythrins, phycoerythrocyanins, phycocyanins, and allophycocyanins, which absorb pink-purple, orange, blue, and red colors, respectively (Esim et al. 2024).

Besides their potential role in light fixing and cyanobacterial photosynthesis, PBPs are also extremely important to humans. They exhibit various biological activities, including antioxidant, anti-inflammatory, anti-aging, antiviral, antibacterial, anticancer, hepatoprotective, and neuroprotective effects, and therefore find notable applications in the cosmetic, pharmaceutical, and nutraceutical industries (Manirafasha et al. 2020; Patel et al. 2022; Esim et al. 2024). To date, several studies have focused on elucidating the antioxidant properties of *Cyanobacteria*-derived PBPs (Table 2)*.* In a similar study, Sonani and et al. (2017) investigated the antioxidant activity and anti-aging potential of phycocyanin purified from *Synechococcus* sp. R42DM. They reported that the phycocyanin exhibited considerable DPPH radical-scavenging and ferric-reducing abilities. Furthermore, the authors noted that phycocyanin alleviated thermal and oxidative stress in C. elegans by scavenging intracellular reactive oxygen species (ROS) molecules, and also improved the physiological behavior and extended the lifespan of *C. elegans*. In a recent study, Suphan et al. (2023) purified and characterized the PBPs from the cyanobacterium *Nostoc* sp. strain SW02, and then assessed their biological activities. The structural analyses confirmed that the PBS extract contained phycoerythrin and phycocyanin. Biological activity assays revealed that the PBS-extract had characteristic antioxidant potential in the DPPH radical-scavenging assay and also showed antibacterial and anticancer properties. In another study, Patel and coworkers (2018) reported that phycocyanin was derived from *Geitlerinema* sp. H8DM demonstrated antioxidant capacities in DPPH and O_2_·^–^ radical-scavenging, ferric ion-chelating, and ferric-reducing power assays. A different research team (Kaur et al. 2019) reported that phycocyanin extracted from the filamentous cyanobacterium *Anabaena fertilissima* exhibited characteristic antioxidant activity against DPPH radicals.

**Table 2:** Antioxidant and other biological activities of bacteria-derived phycobiliproteins

Compound	Bacterium/phylum	Activity	References
Phycocyanin	*Synechococcus* sp. R42DM (*Cyanobacteria*)	DPPH scavenging (70% and 100% respectively for phycocyanin and AA at a concentration of 80 μg/mL) and ferric reducing power activities (In vitro), as well as the reduction of intracellular ROS and the enhancement of lifespan in *C. elegans* (In vivo)	(Sonani et al. 2017)
Phycoerythrin and phycocyanin	*Nostoc sp.* SW02 (*Cyanobacteria*)	DPPH scavenging (IC_50_ values of 30.56 μg/mL and 26.93 μg/mL respectively for phycocyanin and AA, respectively) as well as antibacterial and anticancer activities (In vitro)	(Suphan et al. 2023)
Phycocyanin	*Geitlerinema* sp.H8DM (*Cyanobacteria*)	DPPH (72.85% and 99.82% respectively for phycocyanin and AA at a concentration of 200 μg/mL) and O_2_^·–^ (15.19% and 99.30% respectively for phycocyanin and AA at a concentration of 100 μg/mL) radicals-scavenging and ferric reducing power (85.15% for phycocyanin at a concentration of 150 μg/mL) activities (In vitro)	(Patel et al. 2018)
Phycocyanin	*Anabaena fertilissima* (*Cyanobacteria*)	DPPH radical scavenging activity (Aqueous solution of phycocyanin scavenged 56 nmol of DPPH per mg of phycocyanin while methanolic solution scavenged only 23 nmol of DPPH) (In vitro)	(Kaur et al. 2019)

*DPPH* 2,2-Diphenyl-1-picrylhydrazyl, *O*_*2*_^·−^ superoxide radical and *H*_*2*_*O*_*2*_ hydrogen peroxide

### Mycosporins-like amino acids

Mycosporin-like amino acids (MAAs) are nitrogenous, water-soluble, and colorless biomolecules. They can absorb UV-A and UV-B radiation, stabilize free radicals, and disperse the energy as heat. Various organisms, including lichens, fungi, algae, and cyanobacteria, can synthesize them. The examples of MAAs are mycosporin-glycine, porphyra 334, shinorin, asterina-330, palythene, and palythine. Due to their capacity to absorb radiation and stabilize free radicals, MAAs function as antioxidant and photoprotective compounds in these organisms (Raj et al. 2021; Geraldes and Pinto 2021; Esim et al. 2024).

The MAAs from these organisms exhibit antioxidant and anti-inflammatory activities, and also inhibit collagenase activity. Due to these activities, MAAs can be utilized as UV-absorbing sunscreens and anti-aging agents in cosmetics (Kageyama et al. 2019; Geraldes and Pinto 2021) (Table 3). To date, some studies have been undertaken to evaluate the antioxidant activities of cyanobacteria-derived MAAs (Table 3). For example, in a study by Rastogi and Incharoensakdi (2014), the MAA extract (shinorine and M-307) was prepared from the cyanobacterium *Gloeocapsa* sp. CU-2556 was found to have dose-dependent antioxidant activity in the DPPH assay. In a separate study, Ishihara and co-workers (2017) found that the water extract from the edible cyanobacterium *Nostoc sphaericum* contained different MAAs, namely β-Gal-P334, shinorine, and porphyra-334. They also elucidated that this extract is capable of protecting human keratinocytes (HaCaT) against UV and oxidative stress-induced cell damage. In a study conducted by Kokabi and et al. (2022), the MAAs extracted from the cyanobacterium *Leptolyngbya* sp. were found to comprise nine different MAAs and exhibited antioxidant activity in the ABTS assay. A study conducted by Singh and co-workers (2022) focused on examining the chemical composition and antioxidant potency of the MAAs extracts from two cyanobacteria (*Anabaena* sp. HKAR-7 and *Fischerella* sp. AR-5). The structural analyses revealed that the HKAR-7 extract contained only one type (P-334) of MAAs, whereas three types of MAAs, namely Shinorine, MG-310, and Palythinol, were present in the HKAR-5 extract. In comparison with shinorine, P-334 and MG-310 were determined to possess higher scavenging potency against DPPH and O_2_·^−^ radicals. A study conducted by Browne and et al. (2023) revealed that 8 of 53 Irish marine cyanobacteria possess the capacity to synthesize MAAs. In the study, chemical analyses revealed that eight isolates were capable of synthesizing eight known and four novel MAAs. The in vitro antioxidant activity assays demonstrated that the MAAs of all cyanobacteria possessed DPPH radical-scavenging and ferric-reducing power capacities. In a newly published work (Rastogi et al. 2023), the researchers focused on the radical-scavenging and photoprotective properties of the MAAs extract from the cyanobacterium *Euhalothece* sp.WR7. The structural analyses of the study revealed that the extract contained two different MAAs (mycosporine-2-glycine and Euhalothece-362) and exhibited antioxidant potency in scavenging DPPH radicals. Furthermore, the activity assays indicated that the extract could reduce the production of UV-induced ROS, thereby displaying an efficient photoprotective function.

**Table 3:** Antioxidant and other biological activities of bacteria-derived mycosporins-like amino acids

Compound	Bacterium-Phylum	Activity	References
The extract (shinorine and M-307)	*Gloeocapsa* sp. CU-2556 *(Cyanobacteria)*	DPPH radical scavenging activity (64.3% at an extract concentration of 1.56 mg/mL) (In vitro)	(Rastogi and Incharoensakdi 2014)
β-Gal-P334, shinorine and porphyra-334	*Nostoc sphaericum* *(Cyanobacteria)*	ABTS radical-scavenging activity (IC_50_ value of 17 mM for β-Gal-P334) and UV-protective ability (In vitro)	(Ishihara et al. 2017)
Extract (palythine, palythenic acid, euhalothece-362, palythene, usujirene, palythine serine, asterina-330, palythinol, and palythine-threonine)	*Leptolyngbya* sp. *(Cyanobacteria)*	ABTS radical scavenging activity (54% and 81% for the extract and AA at the dose of 2 mg/mL) (In vitro)	(Kokabi et al. 2022)
Shinorine, MG-310, Palythinol and P-334	*Anabaena* sp. HKAR-7 and *Fischerella* sp. AR-5 *(Cyanobacteria)*	DPPH (90%, 84% and 98% respectively for P-334, MG-310 and AA at the concentration of 100 μL) and O_2_^·–^ (70% and 67% and 98% respectively for P-334 and MG-310 at a concentration of 100 μL) radicals-scavenging, as well as ferric reducing power activity (In vitro*)*	(Singh et al. 2022)
The extract (mycosporine- glutamicol, mycosporine-serinol, mycosporine-taurine, palythine, threonine-sulphate, porphyra-334, usujirene, M-314, M-326)	*Leptolyngbya tenuis* SABC010201, *Phormidium angustissimum* SABC020801, and *Schizothrix* sp. SABC022401 *(Cyanobacteria)*	DPPH radical-scavenging activities from 167.7193 to 764.2105 μM TE g/DW and ferric reducing power activities from 223.05556 to 727.2222 μM TE g/DW (In vitro*)*	(Browne et al. 2023)
Extract containing mycosporine-2 Glycine (M2G) and Euhalothece-362)	*Nostoc sphaericum* *(Cyanobacteria)*	DPPH radical scavenging (59.95% at the extract concentration of 1 mg/mL) and the reduction of UV-induced ROS (In vitro)	(Rastogi et al. 2023)

*ABTS* 2,2'-azino-bis(3-ethylbenzothiazoline-6-sulfonic acid), *DPPH* 2,2-Diphenyl-1-picrylhydrazyl, *O*_*2*_^·–^ superoxide radical and *ROS* reactive oxygen species. *AA* Ascorbic acid represents the control compound used in antioxidant activity assays

### Peptides

Bioactive peptides are low-molecular-weight protein fragments composed of specific amino acid sequences. They exhibit diverse bioactive properties, including antioxidant, antiviral, immunomodulatory**,** anti-inflammatory, antimicrobial, antiparasitic, anticancer, anti-obesity, anti-adipogenic, anti-hypertensive, antithrombotic, and antidiabetic activities (Akbarian et al. 2022; Rizwan et al. 2023; Arslan et al. 2023).

Bioactive peptides are primarily prepared from the proteins of plants and animals using various hydrolysis methods, including enzymatic hydrolysis, acid-alkaline hydrolysis, and microbial fermentation-based hydrolysis. On the contrary, specific bioactive peptides can be naturally synthesized by bacteria, archaea, fungi, algae, animals, and plants (Arslan et al. 2023, 2024). Natural peptides synthesized by bacteria are classified into two main groups based on their biosynthesis type: ribosomally synthesized peptides and non-ribosomally synthesized peptides (Arslan et al. 2024).

Lipopeptides, also known as microbial surfactants, are produced extracellularly or as a part of the cell membrane by several bacterial and fungal species. In the bacteria, these peptides are mainly synthesized in a ribosome-independent manner by non-ribosomal peptide synthetases (NRPS), which deliver one amino acid to the growing peptide chain (Roongsawang et al. 2010; Théatre et al. 2021; Muangkaew et al. 2023). *Members of Bacillus, Pseudomonas, and Streptomyces are potential producers of lipopeptides (*Roongsawang et al. 2010; Seo et al. 2022). Lipopeptides are recognized for their potent antimicrobial properties. Furthermore, they exhibit strong antioxidant activities (Table 4). For example, Jemil and coworkers (2017) reported that the lipopeptides from *Bacillus methylotrophicus* DCS1 exhibit in vitro antioxidant activities in DPPH radical-scavenging, ferric-reducing power, and lipid peroxidation inhibition assays. They also found that the lipopeptides exhibited significant antibacterial, antifungal, and anti-adhesive activities. In another study, Ayed et al. (2015) demonstrated that the lipopeptides from *B. mojavensis* A21 exhibited in vitro antioxidative potential, as evidenced by DPPH radical-scavenging activity, metal-reducing power, and lipid peroxidation inhibition assays. Additionally, the in vivo experiments demonstrated that the lipopeptide-gel formulation can accelerate wound healing in a rat model, resulting in complete wound closure. A study performed by Rahman and co-workers (2018) demonstrated that the peptide YD1, purified from *Bacillus amyloliquefaciens* CBSYD1, exhibited radical-scavenging (DPPH and ABTS radicals) and ferric-reducing power activities, as well as antimicrobial efficiency. Moreover, the peptide was found to enhance the activities of antioxidant enzymes and reduce the levels of intracellular NO^·^ and ROS in RAW 264.7 cells exposed to LPS-induced oxidative stress. In their work, Seo and et al. (2022) focused on evaluating the biological activities of the lipodepsipeptides (cystargamides B, C, and D) isolated from the marine actinomycete *Streptomyces* sp. JMS132. The structural analyses revealed that the peptides are made up of six different amino acids with an epoxy fatty acid side chain in their chemical structure. The in vitro assays demonstrated that the lipopeptides possess antioxidant potency, as evidenced by their ability to scavenge DPPH and ABTS radicals. In a study conducted by Dussert and et al. (2022), the antiradical and antioxidant capacities of lipopeptides (surfactin, mycosubtilin, and plipastatin/fengycin) from *B. subtilis* strains were evaluated. All the peptides were found to exhibit antioxidant activity against OH·, O_2_·^–^, and DPPH radicals; however, the scavenging potential of plipastatin was higher than that of the others. Ren and coworkers (2022) demonstrated that epichlicin, a cyclic lipopeptide derived from the fermentation broth of *B. amyloliquefaciens* SJ100001, exhibits antioxidant properties, including DPPH radical-scavenging and ferric-reducing capabilities, as well as antifungal properties. A different research group (Khan et al. 2020) reported that the peptide purified from the culture broth of *Bacillus velezensis* exhibits strong antioxidant potency in radical-scavenging assays (DPPH and ABTS) and metal-reducing assays (ferric and cupric-reducing). Moreover, the peptide was found to exhibit significant antioxidant activity by enhancing the activities of antioxidant enzymes and reducing the levels of intracellular NO· and ROS in RAW 264.7 cells subjected to LPS-induced oxidative stress. In a recently conducted study (Dai et al. 2024), researchers aimed to optimize surfactin production from SOPC5 in a soybean meal-based medium and then evaluate the in vitro antioxidative potential of the produced surfactin. Optimization experiments revealed that soybean concentration, mineral content, temperature, and incubation time significantly affected surfactin production. In vitro antioxidant assays demonstrated that the purified surfactin is capable of scavenging DPPH and ABTS radicals.

**Table 4:** Antioxidant and other biological activities of bacteria-derived peptides

Compound	Bacterium-Phylum	Activity	References
Lipopeptides	*Bacillus mojavensis* A21*(Firmicutes)*	DPPH radical-scavenging (65% at the concentration of 1 mg/L), metal reducing power (2.0 at OD_700_ nm at a dose of 10 mg/mL), and lipid peroxidation inhibition activity (greater than 70% after 7 days of incubation at concentrations ≥ 10 mg/mL) (In vitro), as well as wound healing activity in a rat model (In vivo)	(Ayed et al. 2015)
Lipopeptides (DCS1)	*B. methylotrophicus* DCS1 *(Firmicutes)*	DPPH radical scavenging (80.6% and 100% for DCS1 and the control BHA at a concentration of 1 mg/mL), lipid peroxidation inhibition (about 76.8% at a concentration of 0.1 mg/mL) and ferric reducing power (an absorbance of 3.0 for both DCS1 and BHA at a concentration of 2.0 mg/mL) activities as well as antibacterial, antifungal and anti-adhesive activities (In vitro)	(Jemil et al. 2017)
Peptides	*B. amyloliquefaciens* CBSYD1 *(Firmicutes)*	DPPH and ABTS radical scavenging and ferric reducing power activities, as well as the increases in the activities of SOD1, CAT, and GPx-1 and the decreases in the levels of intracellular NO^·^ and ROS (In vitro). The peptide also exhibed antimicrobial efficiency against various pathogens including multi drug resistant bacteria	(Rahman et al. 2018)
Lipopeptides (surfactin, mycosubtilin, and plipastatin)	*B. subtilis* *(Firmicutes)*	Plipastatin and surfactin/plipastatin mixture exhibited the scavenging activities of 18.48% and 22.88% against DPPH radical. Against H_2_O_2_, ·OH and O_2_^·–^ radicals, the scavenging activities were 6, 21, and 3% for surfactin, 19, 9, and 15% for mycosubtilin, 21, 18, and 59% for plipastatin, 21, 31, and 61% for the mixture of surfactin/plipastatin, and 13, 16, and 15% for the mixture of surfactin/mycosubtilin, respectively (In vitro)	(Dussert et al. 2022)
Lipopeptide (Epichlicin)	*B. amyloliquefaciens* SJ100001	DPPH scavenging activity of 94.99% of epichlicin and its ferric-reducing ability reaching half of that of the positive control AA, at a concentration of 4.0 mg/mL (In vitro). Besides, antifungal activity against *F. oxysporum* SJ300024 (In vitro)	(Ren et al. 2022)
Peptide	*B. velezensis*	ABTS (68% and 77% for peptide and AA), DPPH (17–69% for peptide and 32–85% for AA), O_2_^·–^ (6–67% for peptide and 29–82% for AA), ferric-reducing power acticity (72.73% for peptide and AA). The enhancement of antioxidant enzyme activities (SOD1, CAT, and GPx-1) and the reduction of intracellular NO^·^ and ROS in RAW 264.7 cells (In vitro)	(Khan et al. 2020)
Surfactin	*B. subtilis*	DPPH and ABTS radicals-scavenging activities (IC_50_ values of 1.275 and 0.73 mg/mL for surfactin, respectively) (In vitro)	(Dai et al. 2024)
Bacteriocin	*Lactococcus lactis*	DPPH radical scavenging activity (EC_50_ value of 12.5 μg/mL) as well as antimicrobial and anti-biofilm activities (In vitro)	(Krishnamoorthi et al. 2022)
ALC101 and ALC102 (Bacteriocins)	*Paenibacillus polymyxa* and *Enterococcus faecium*	Scavenging activities towards DPPH (85% for ALC101 and ALC102) and ABTS (85.5% for ALC101 and ALC102 at the concentration of 100 μg/mL) (In vitro)	(Krishna et al. 2021)
Bacteriocin	*Bifidobacterium* sp.	Antioxidant (DPPH radical-scavenging activity of 82.55% at 120 μg/mL), antibacterial (against *E. coli* and *B. subtilis*) and anti-inflammatory activities (In vitro)	(Priyanka et al. 2024)
Lipodepsipeptides (Cystargamides B, C and D)	*Streptomyces* sp. JMS132 (*Actinobacteria*)	DPPH and ABTS radicals scavenging activities (53% and 100% respectively at a concentration of 200 μg/mL) (In vitro)	(Seo et al. 2022)

*ABTS* 2,2'-azino-bis(3-ethylbenzothiazoline-6-sulfonic acid; DPPH, 2,2-Diphenyl-1-picrylhydrazyl, *ROS* reactive oxygen species, ·*OH* hydroxyl radical, *O*_*2*_^·–^ superoxide radical, *H*_*2*_*O*_*2*_ hydrogen peroxide, *NO*^·^ nitric oxide radical, *SOD* superoxide dismutase, *CAT* catalase, *GPx* glutathione peroxidase and *MDA* malondialdehyde, *BHA* Butylated hydroxyanisole and *AA* ascorbic acid represent the control compounds used in antioxidant activity assays

Bacteriocins are examples of ribosomally synthesized peptides produced by bacteria. They are synthesized by both Gram-negative and Gram-positive bacteria, mainly by lactic acid bacteria such as *Lactobacillus*, *Lactococcus*, *Streptococcus*, *Enterococcus,* and *Pediococcus*. Due to their potent antimicrobial properties, they are used in the preparation of drug formulations and as food preservatives (Arslan et al. 2024). Furthermore, some of them have been documented to exhibit antioxidant activities (Table 4). For instance, a study by Krishnamoorthi et al. (2022) examined the biochemical properties and biological activities of the bacteriocin produced by the *Lactococcus lactis* strain CH3. They have determined that the purified bacteriocin has a molecular weight of 3.5 kDa and consists of six amino acids. They also found that the bacteriocin possesses potent antimicrobial and anti-biofilm activities against human pathogens and exhibits high DPPH radical-scavenging potential. A different research group (Krishna et al. 2021) demonstrated that bacteriocins from *Paenibacillus polymyxa* and *Enterococcus faecium* exhibited in vitro antioxidant properties in DPPH and ABTS radical-scavenging assays, as well as metal-reducing capabilities. A recently published study (Priyanka et al. 2024) investigated the potential biological activities of a bacteriocin isolated from *Bifidobacterium* sp. The in vitro assays clarified that the purified bacteriocin had antioxidant (against DPPH radicals), antibacterial, and anti-inflammatory activities.

### Polysaccharides

Polysaccharides are polymers of carbohydrates that consist of long chains of monosaccharides. They are widely distributed in bacteria, fungi, algae, humans, lichens, animals, and plants (Arslan et al. 2023, 2024; Esim et al. 2024). According to their localization, bacteria-derived polysaccharides are divided into two main groups: capsular polysaccharides (CPS) and exopolysaccharides (EPS). CPSs are known to be linked to the cell surface, while EPSs are released on the cell surface without attachment to the cell (Cescutti 2010).

Bacteria-derived EPSs exhibit diverse biological activities, including antioxidant, antimicrobial, anticancer, anti-aging, cholesterol-lowering, antiviral, and anti-obesity properties, and therefore have both medicinal and biotechnological importance (Ayyash et al. 2020; Kaur and Dey 2022; Arslan et al. 2024). To date, various research groups have attempted to evaluate the antioxidant capacities of polysaccharides derived from different bacterial phyla (Table 5). In a study, Manivasagan et al. (2013) evaluated the antioxidant capacity of the EPS from the marine *Streptomyces violaceus* MM72. They found that the EPS exhibits strong radical-scavenging (DPPH and O_2_·^–^ radicals) and metal-chelating activities, as well as moderate inhibition of lipid peroxidation. Elnahas and coworkers (2017) examined the structural properties and antioxidant potency of the EPS from *Streptomyces* sp. MOE6. Based on structural analysis, the EPS was determined to be a heteropolysaccharide made up of glucose and mannose monomers. The in vitro antioxidant activity assays indicated that the EPS exhibits strong scavenging activities against DPPH and ·OH radicals, and is also capable of chelating ferrous ions. The cytotoxicity assay of the study demonstrated that EPS caused inhibition of the proliferation of human breast cancer cells. Vinothini and coworkers (2019) examined the structural properties and biological activities of EPS obtained from the actinobacterium *S. griseorubens* GD5. The structural analyses revealed that EPS is a heteropolysaccharide composed primarily of arabinose, glucose, galactose, mannose, and xylose monomers. Based on the data from the in vitro antioxidant activity assays, the EPS was found to possess strong DPPH radical-scavenging and ferric-reducing potential. Xiong and et al. (2020) evaluated the antioxidant activities of two EPSs isolated from the endophytic actinobacterium *Glutamicibacter halophytocola* KLBMP 5180. They found that the EPSs exhibited moderate scavenging activity against DPPH and O_2_·^–^ radicals, as well as strong scavenging activity against ·OH radicals. They also found that the EPSs exhibited strong antioxidant activity in the lipid peroxidation inhibition assay but had a low antioxidant potential in the ferric-reducing power assay. Overall, the authors concluded that EPSs have a moderate antioxidant potential. In a recent study, Mahmoud and et al. (2023) evaluated the antioxidant and anti-Alzheimer’s potential of the EPS from the marine bacterium *Streptomyces* sp. NRCG4. The biochemical analysis clarified that the EPS is an acidic polysaccharide composed of mannuronic acid, glucose, mannose, and rhamnose. The in vitro antioxidant activity assays indicated that the EPS had a high capacity to scavenge DPPH, ABTS, and NO· radicals, as well as moderate capacities to chelate ferrous ions and inhibit lipid peroxidation. The biological activity assays also elucidated that the EPS had anti-cholinesterase, anti-tyrosinase, and anti-inflammatory activities. Overall, due to its anti-cholinesterase, anti-tyrosinase, anti-inflammatory, and antioxidant abilities, EPS is suggested to be used as a promising natural metabolite for treating Alzheimer’s disease. In a study conducted by Mohamed and et al. (2023), the structural properties and biological activities of the EPS from the actinobacterium *S. rochei* strain OF1 were investigated. The researchers determined that EPS is a heteropolysaccharide consisting of different monomers, including mannuronic acid, glucuronic acid, xylose, and fructose. They found that EPS had in vitro antioxidant potential, capable of scavenging DPPH radicals, and exhibited antimicrobial effectiveness against bacteria and fungi. Furthermore, they elucidated that the EPS could alleviate carrageenan-induced arthritis in the in vivo rat model by exhibiting anti-inflammatory activity. In a recent study, Li and co-workers (2023) isolated the EPS from the fermentation broth of the actinobacterium *Rhodococcus qingshengii* QDR4-2 and examined its chemical structure and antioxidant potential. They found that the EPS was a heteropolysaccharide composed of mannose and glucose monomers with a molecular weight of 9.450 × 10^5^ Da and could scavenge DPPH, ABTS, ·OH, and O_2_·^–^ radicals. In a recently published study (El Awady et al. 2024), the researchers sought to elucidate the structural and bioactive properties, including antioxidant and anti-inflammatory activities, of the extracellular polymeric substance (EPS) from the halophilic actinobacterium *Kocuria* sp. clone Asker4. They found that EPS is a heteropolysaccharide composed of three main monomers (fructose, glucuronic acid, and xylose) and exhibits in vitro antioxidant activity. They also revealed that carrageenan-induced paw edema reduced the antioxidant capacity and caused inflammation in the in vivo rat model, while the EPS treatment could prevent these detrimental effects. Kim and coworkers (2022) examined the chemical structure and biological activities of the EPS from the proteobacterium *Lysobacter* sp. MMG2. The EPS was ascertained to be a heteropolysaccharide composed of mannose, glucose, galactose, arabinose, xylose, and rhamnose monomers. The EPS was found to exhibit inhibitory effects against biofilm-forming pathogenic bacteria and also possesses antioxidant activity, scavenging DPPH radicals. A previous study (Dhanya et al. 2022) investigated the structural properties and biological activity of the extracellular polymeric substance (EPS) from the marine proteobacterium *Pseudoalteromonas* sp. YU16-DR3A. The study clarified that EPS is a heteropolysaccharide composed of fucose, erythritol, ribose, and glucose monomers, and it displays in vitro antioxidant capacity by scavenging DPPH and NO^·^ radicals. Ye et al. (2016) reported that the selenium-containing exopolysaccharide (Se-EPS) from *Pseudomonas* PT-8 exhibited strong scavenging effects on DPPH, ·OH, and O_2_·^–^ radicals. In another study, Belhaj et al. (2017) elucidated that the EPS purified from the culture broth of *Phormidium versicolor* was a sulfated heteropolysaccharide composed of different monomers, namely arabinose, xylose, ribose, rhamnose, N-acetylglucosamine, galactose, glucose, mannose, glucuronic acid, and saccharose. They found that the EPS could scavenge DPPH and ·OH radicals and also exhibit ferric-reducing power and β-carotene-bleaching activities. Tiwari and co-workers (2019) analyzed the structural properties and antioxidant potential of two EPSs extracted from *Anabaena* CCC 745. The EPSs were determined to be negatively charged heteropolysaccharides composed of glucose, xylose, rhamnose, and glucuronic acid. The in vitro assays revealed that the EPSs exhibited significant antioxidant activity in scavenging H_2_O_2_ radicals. Gongi et al. (2022a) investigated the physicochemical and antioxidant properties of the extracellular polymeric substance (EPS) from the filamentous cyanobacterium *Leptolyngbya* sp. IkmLPT16. The structural analyses revealed that the EPS is a heteropolysaccharide composed of mannose, arabinose, glucose, rhamnose, and uronic acid monomers. The elemental analysis of the EPS displayed the presence of sulfate groups. The EPS demonstrated potent antioxidant capacity by scavenging radicals, particularly DPPH and ·OH radicals, as well as chelating ferrous ions. In a separate study, the same research group (Gongi et al. 2022b) investigated the structural properties and antioxidant potential of EPSs from the thermophilic cyanobacterium *Gloeocapsa gelatinosa*. They found that the EPSs were sulfated heteropolysaccharides composed of different monomers, mainly mannose and uronic acids, and exhibited in vitro antioxidant activities, including DPHH radical scavenging, *β*-carotene bleaching (linoleic acid oxidation), and iron-chelating power.

**Table 5:** Antioxidant and other biological activities of bacteria-derived polysaccharides

Compound	Bacterium/phylum	Activity	References
EPS	*Streptomyces* sp. NRCG4 (*Actinobacteria*)	The scavenging activities of EPS, BHT and AA (at a concentration of 400 μg/mL) were 88.26%, 98.32% and 100% against DPPH, 98.69%, 100% and 100% against ABTS, 81.27%, 97.75% and 98.11% against O_2_^·^, and 58.64%, 93.27% and 95.88% against NO^·^, respectively. IC_50_ values of EPS, BHT and ascorbic acid were 150.34, 85.17 and 88.11 in metal chelation assay and 190.45, 18.33 and 19.65 in lipid peroxidation inhibition assay, respectively. Besides, anti-cholinesterase, anti-tyrosinase and anti-inflammatory (COX-1 and COX-2 inhibition) activities (In vitro)	(Mahmoud et al. 2023)
EPS	*Streptomyces* sp. MOE6 (*Actinobacteria*)	DPPH and ·OH radicals-scavenging activities (37.8% and 26% at a concentration of 4 mg/mL) and ferrous-chelating power activities (92% and 98.4% for EPS and EDTA–Na^+^ at a concentration of 2 mg/mL) as well as anticancer activity against human breast cancer cells (MDA-MB-231) (In vitro)	(Elnahas et al. 2017)
EPS	*S. violaceus* MM72 (*Actinobacteria*)	DPPH (IC_50_ value of 76.38 mg/mL) and O_2_^·^ (IC_50_ value of 67.85 mg/mL) radicals-scavenging, lipid peroxidation inhibition (73.21% and 92.76% for EPS and AA at a concentration of 1 mg/mL), metal chelating and ferric reducing power activities (In vitro)	(Manivasagan et al. 2013)
EPS	*S. griseorubens* GD5 (*Actinobacteria*)	DPPH radical scavenging (70.29% and 83.12% respectively for EPS and AA at the concentration of 1 mg/mL), ferric reducing power (1.033 and 1.525 respectively for EPS and AA at 1 mg/mL concentration) and metal chelating (85.8% and 97.2% respectively for EPS and EDTA at a dose of 1 mg/mL) activities (In vitro)	(Vinothini et al. 2019)
EPS	*S.rochie* OF1 (*Actinobacteria*)	DPPH radical scavenging (92.06%), antimicrobial (against *S. aureus, E. coli*, MRSA, *K. pneumoniae* and *C. albicans*) and anti-inflammatory (TNF-α and COX2 inhibition) activities (In vitro)	(Mohamed et al. 2023)
EPS (EPS-1 and EPS-2)	*Glutamicibacter halophytocola* KLBMP 5180 (*Actinobacteria*)	Scavenging activities against DPPH (25.97% and 23.17% for EPS-1 and EPS-2 at the concentrations of 3.0 g/L and 2.04 g/L, respectively), ·OH (61.80% and 58.75% for EPS-1 and EPS-2 at the concentrations of 0.8 g/L and 1.0 g/L, respectively) and O_2_^·^ (61.80% and 58.75% for EPS-1 and EPS-2 at the concentrations of 0.8 g/L and 1.0 g/L, respectively), as well as lipid peroxidation inhibition activities (25.26% and 23.47% for EPS-1 and EPS-2 at the concentrations of 2 g/L and 3 g/L, respectively) (In vitro)	(Xiong et al. 2020)
EPS	*Rhodococcus qingshengii* QDR4-2 (*Actinobacteria*)	DPPH, ABTS, ·OH and O_2_^·–^ radicals-scavenging activities (49.09%, 45.77%, 30.24%, and 39.18%, respectively, at 3 mg/mL concentration) (In vitro)	(Li et al. 2023)
EPS	*Kocuria* sp. (*Actinobacteria*)	DPPH scavenging activity (94.13% at 100 µg/mL concentration) (In vitro) as well as the increased antioxidant capacity (the enhancement of GSH, CAT and SOD activites and the reduction of lipid peroxidation and ROS levels) and anti-inflammatory (the reduction of NO^·^, COX-2 and IL-6 levels) activity (In vivo)	(El Awady et al. 2024)
EPS	*Lysobacter* sp. MMG2 (*Proteobacteria*)	DPPH radicals scavenging activity (at the concentration increased to 4 mg/mL, 89.25% and 96.5% for EPS and AA, respectively (In vitro)	(Kim et al. 2022)
EPS	*Pseudoalteromonas* sp. YU16-DR3A (*Proteobacteria*)	DPPH and NO^·^ radicals-scavenging activities (41.3% and 27.6% respectively at a concentration of 5 mg/mL) (In vitro)	(Dhanya et al. 2022)
(Se-EPS)	*Pseudomonas* PT-8 (*Proteobacteria*)	Scavenging activities against DPPH (53.53% for Se-EPS, which is around 1/2 of the scavenging capacity of AA), ·OH (84.42% and 92.01% for Se-EPS and AA), and O_2_^·–^ (93.43% and 97.67% for Se-EPS and AA). The reducing power potency of Se-EPS was about 50% equivalency of AA when at a concentration of 3 mg/mL (In vitro)	(Ye et al. 2016)
EPS	*Phormidium versicolor* (*Cyanobacteria*)	Scavenging activities against DPPH (57.3% and 91.12% respectively for EPS and AA at the concentration of 625 μg/mL) and ·OH (64.7% for EPS at a concentration of 1.25 mg/mL). Besides, ferric reducing power (0.84 mM/L and 0.93 mM/L respectively for EPS and AA at a concentration of 2.5 mg/mL) and β-carotene bleaching (100% for EPS at a concentration of 1.25 mg/mL) activities (In vitro)	(Belhaj et al. 2017)
EPS	*Leptolyngbya* sp. IkmLPT16 (*Cyanobacteria*)	Scavenging activities DDPH· (IC_50_ values of 4 mg/mL and 10 mg/mL for EPS and AA, respectively) and ·OH (IC_50_ values of 10 mg/mL and 20 mg/mL for EPS and AA, respectively), as well as ferrous chelating activity (IC_50_ of 40 mg/mL and 60 mg/mL for EPS and EDTA, respectively) (In vitro)	(Gongi et al 2022a)
EPS	*Gloeocapsa gelatinosa* (*Cyanobacteria*)	DPPH scavenging (IC_50_ values of 0.2 and 0.6 g/L for EPS and AA, respectively), *β*-carotene bleaching effect (IC_50_ value of 0.5 g/L for both EPSs and AA), and metal chelating (IC_50_ values of 0.4 and 0.6 g/L for EPS and ETDA) activity (In vitro)	(Gongi et al. 2022b)
EPS	*Lactobacillus plantarum* KX041 *(Firmicutes)*	The scavenging activities against DPPH (IC_50_ value of 1.4 mg/mL), ABTS (IC_50_ value of 0.2 mg/mL), ·OH (IC_50_ value of 1.7 mg/mL) and O_2_^·–^ (IC_50_ value of 5.6 mg/mL), as well as ferric reducing power activity (0.641 at a concentration of 8 mg/mL) (In vitro)	(Wang et al. 2017)
EPS	*Lactobacillus fermentum* S1 *(Firmicutes)*	Ferric reducing power (118.70 μM) and the radical-scavenging activities against ABTS (99.53%), DPPH (83.05%), and ·OH (68.93%) at a concentration of 4 mg/mL (In vitro). Besides, the increases in SOD and total antioxidant activities and the decrease in MDA level (In vivo)	(Wang et al. 2019)
EPS	*Enterococcus faecium* MS79 *(Firmicutes)*	ABTS and DPPH radicals-scavenging activities (85% and 44% at a concentration of 10 mg/mL, respectively) and anticancer effectiveness against colon and breast cancer cell lines (In vitro)	(Ayyash et al. 2020)
EPS	*Pediococcus acidilactici* NCDC 252 *(Firmicutes)*	Scavenging activities against DPPH (54.9% at a concentration of 10 mg/mL) and H_2_O_2_ (93% at a concentration of 10 mg/mL) radicals and the ferric reducing power capacity (0.240 and 1.249 for EPS and AA at a concentrtion of 10 mg/mL). Basides, anticancer effectiveness against human colon cancer cell line (HCT116) (In vitro)	(Kumar et al. 2020)
EPS (EPS1, EPS2 and EPS3)	*Streptococcus thermophiles* *(Firmicutes)*	DPPH (47.11%, 70.18%, and 41.79% for EPS1, EPS2 and EPS3 at a maximum concentration of10 mg/mL, respectively), ·OH (a higher scavenging activity of 42.29% for EPS2 when compared to EPS1 and EPS2) and O_2_^·–^ (49.47%, 58.30%, and 52.63% for EPS1, EPS2 and EPS3 at a maximum concentration of 10 mg/mL, respectively) radicals-scavenging activities as well as the reduction of intracellular ROS in intestinal epithelial cells (In vitro)	(Xu et al. 2023)
EPS (EPS1-EPS10)	*S. thermophiles* DSM 24731, *L. delbrueckii* ssp. *bulgaricus* DSM 20081 T, and *Limosilactobacillus fermentum* DSM 20049 *(Firmicutes)* and *Bifidobacterium longum* ssp. *longum* DSMS 200707 (*Actinobacteria*)	DPPH radical-scavenging (33.3–77% for EPS1-EPS10 at a concentration of 20 mg/mL) and total antioxidant (0.75–2.5% for EPS1-EPS10 at a concentration of 20 mg/mL) activities as well as anticancer effectiveness against human cancer cell lines (MCF7, CaCO-2, and HepG2) (In vitro)	(Khalil et al. 2022)
EPS	*Bacillus cereus* SZ-1 *(Firmicutes)*	DPPH (54.0% at a concentration of 3 mg/mL), ·OH (50.4% at a concentration of 2 mg/mL), O_2_^·–^ (50.1% at a concentration of 1 mg/mL) radicals-scavenging and ferric-reducing power activities. Besides, the increases in GSH level and CAT activity, and the reduction in MDA level in PC12 cells exposed to H_2_O_2_ (In vitro)	(Zheng et al. 2016)
EPS	*B. velezensis* SN-1 *(Firmicutes)*	Scavenging activities of 63%, 63.3% and 60.6% against DPPH, ABTS and ·OH at a concentration of 8 mg/mL. A scavenging activity of 1243.4 μmol TE/g against O_2_^·–^ when used at a concentration of 0.4 mg/mL (In vitro)	(Cao et al. 2020)
EPS	*B. xiamenensis* RT6 *(Firmicutes)*	Scavenging activities against DPPH (around 65% in the concentration range of 0.1 to 10 mg/mL), ·OH (100% at a concentration of 2.5 mg/mL) and O_2_^·–^ (39.4% at a concentration of 10 mg/mL). Besides, the reduction of intracellular ROS content in HeLa cells (In vitro)	(Huang-Lin et al. 2022)
EPS	*B. amyloliquefaciens* RT7 *(Firmicutes)*	Scavenging activities against DPPH (67% and 82% for EPS and AA at a concentration of 7.5 mg/mL), ·OH (90% and 98% for EPS and AA at a concentration of 5 mg/mL) and O_2_^·–^ (96.5% and 100% for EPS and AA at a concentration of 0.25 mg/mL). Besides, the reduction of H_2_O_2_-induced intracellular ROS in HeLa cells (In vitro)	(Sánchez-León et al. 2023)
EPS (EPS-1 and EPS-2)	*Geobacillus* sp. WSUCF1 *(Firmicutes)*	Scavenging activities against DPPH (greater than 80% and greater than 40% respectively for EPS-1 and EPS-2 at a concentration of 10 mg/mL), O_2_^·–^ (greater than 80% and about 40% respectively for EPS-1 and EPS-2 at a concentration of 10 mg/mL), and ·OH (greater than 30% and greater than 80% respectively for EPS-1 and EPS-2 at a concentration of 10 mg/mL) (In vitro)	(Wang et al. 2021)
EPS	*Lysinibacillus sphaericus* Ya6 *(Firmicutes)*	Scavenging activities against DPPH (72.54% at the concentration of 2.5 mg/mL) and O_2_^·–^ (14.97% at a concentration of 0.5 mg/mL) (In vitro)	(Yang et al. 2022)
EPS	*L. fusiformis* KMNTT-10 *(Firmicutes)*	DPPH, ABTS, and NO^·^ radicals scavenging activities (65.21%, 66.17% and 62.84% respectively at a concentration of 3 mg/mL) (In vitro)	(Mathivanan et al. 2021)

*ABTS* 2,2'-azino-bis(3-ethylbenzothiazoline-6-sulfonic acid, *DPPH* 2,2-Diphenyl-1-picrylhydrazyl, *ROS* reactive oxygen species, ·*OH* hydroxyl radical, *O*_*2*_^·–^ superoxide radical, *H*_*2*_*O*_*2*_ hydrogen peroxide, *NO*^·^ nitric oxide radical, *SOD* superoxide dismutase, *CAT* catalase, *GSH* glutathione, *MDA* malondialdehyde, *EPS* exopolysaccharide and *Se-EPS* selenium-containing exopolysaccharide. *BHT* Butylated hydroxytoluene and *AA* ascorbic acid represent the control compounds used in antioxidant activity assays

Lactic acid bacteria (LAB) in the phylum *Firmicutes* are considered good sources of polysaccharides. LAB-derived polysaccharides are widely used in the food industry to enhance the viscosity, texture, and stability of foods. Moreover, they exhibit antioxidant, antibacterial, cholesterol-lowering, and prebiotic properties, and therefore find diverse applications in the cosmetic, pharmaceutical, and medical industries (Saadat et al. 2019; Korcz and Varga 2021; Prete et al. 2021). To date, different studies have been conducted to assess the antioxidant potential of LAB-derived EPSs. In such a study, Wang et al (2017) focused on investigating the structural properties and antioxidant capacity of the EPS from *Lactobacillus plantarum* KX041. The structural analyses revealed that the EPS was a heteropolysaccharide with a molecular weight of 38.67 kDa, primarily composed of arabinose, mannose, glucose, and galactose monomers. The EPS was found to possess a strong antioxidative capacity in ABTS, DPPH, ·OH, and O_2_·^–^ radical-scavenging assays, as well as ferric-reducing power. In another study, Wang et al. (2019) analyzed the structure and antioxidant capacity of a cell-bound exopolysaccharide (c-EPS) from *Lactobacillus fermentum* S1. The structural analyses revealed that c-EPS was a heteropolysaccharide composed of mannose, rhamnose, glucose, and galactose monomers, with an average molecular weight of 19 × 10^5^ Da. Based on the data obtained from radical-scavenging (ABTS, DPPH, ·OH radicals) and ferric-reducing power assays, the c-EPS was found to have in vitro antioxidant potency. Furthermore, the in vivo analyses revealed that the c-EPS could increase SOD and total antioxidant activities and decrease the MDA level *in C. elegans.* Ayyash and et al. (2020) studied the structural properties and biological activities of the EPS purified from *Enterococcus faecium* MS79. They found that the EPS is composed of three monomers (arabinose, mannose, and glucose), exhibits antioxidative capacity in DPPH and ABTS radical-scavenging assays, and also possesses anticancer and antibacterial properties. In a study, Kumar et al. (2020) investigated the biological activities of EPS isolated from Pediococcus acidilactici NCDC 252, finding that the EPS exhibited vigorous antioxidant activities in DPPH and H2O2 radical-scavenging assays, as well as an anti-proliferative effect on the human colon cancer cell line. In another study, Khalil and et al. (2022) focused on the bioactive properties of EPSs purified from the culture supernatants of lactic acid bacteria (LAB) (*Firmicutes*) and bifidobacteria (Bb) (*Actinobacteria*). DPPH radical-scavenging and total antioxidant assays revealed that the EPSs of LAB (*Streptococcus thermophiles* DSM 24731, *Lactobacillus delbrueckii* ssp. *bulgaricus* DSM 20081 T, and *Limosilactobacillus fermentum* DSM 20049) exhibited higher antioxidant potential than those of Bb (*Bifidobacterium longum* ssp. *longum* DSM 200707). The cytotoxicity assays showed that the EPS of *L. delbrueckii* ssp. *bulgaricus* DSM 20081 exhibited a higher cytotoxic potential against cancer cell lines compared to that of other bacteria. A recent study by Xu and co-workers (2023) investigated the antioxidant activity of three different EPS components (ST-ESP1, ST-EPS2, and ST-EPS3) from *Streptococcus thermophilus*. The components were determined to consist of mannose, glucuronic acid, galacturonic acid, glucose, and N-acetylglucosamine monomers. The in vitro antioxidant assays revealed that all the components possessed the ability to scavenge DPPH, ·OH, and O_2_·^−^ radicals, as well as to reduce ferric ions. However, ST-EPS2 showed higher radical-scavenging and ferric-reducing potential compared to the others. ST-EPS2 was also found to decrease ROS accumulation in intestinal epithelial cells (NCM 460) exposed to H_2_O_2_.

Other genera of the phylum *Firmicutes*, such as *Bacillus*, *Geobacillus*, and *Lysinibacillus*, are also capable of producing EPS with antioxidant activity. For example, a previous study (Zheng et al. 2016) revealed that the EPS from the endophytic bacterium *B. cereus* SZ-1 can scavenge ·OH and O_2_·^–^ radicals and exhibit ferric-reducing power activity. Furthermore, the study demonstrated that the EPS was able to protect PC12 cells against H_2_O_2_ toxicity by increasing the GSH level and catalase activity, and by lowering the MDA level. A different research group (Cao et al. 2020) demonstrated that a capsular polysaccharide (CPS) from *B. velezensis* SN-1 exhibits dose-dependent scavenging capabilities against DPPH, OH·, ABTS, and O_2_·^–^ radicals. In another study (Wang et al. 2021), the researchers looked into the antioxidant activities and structural properties of EPSs purified from the thermophilic bacterium *Geobacillus* sp. strain WSUCF1. The structural analyses revealed that EPS-1 is a glucomannan, primarily composed of mannose and glucose units, whereas EPS-2 consists of only mannan monomers. The in vitro antioxidant activity measurements revealed that EPS-1 exhibited a higher scavenging potential against DPPH and O_2_·^–^ radicals, whereas EPS-2 demonstrated a higher potential against ·OH radicals. In a study performed by Huang-Lin et al. (2022), the researchers examined the antioxidant activity and structural properties of EPS derived from the *B. xiamenensis* RT6 strain. The structural analyses revealed that the EPS is a heteropolysaccharide composed of glucose, mannose, and galactose monomers. The antioxidant activity assays showed that the EPS can scavenge radicals (DPPH, ·OH, and O_2_·^–^) and reduce intracellular ROS content in HeLa cells. A previous study (Yang et al. 2022) revealed that the EPS from *Lysinibacillus sphaericus* Ya6 is a heteropolysaccharide composed of mannose and glucose monomers, with a molecular weight of 89.62 kDa. The in vitro antioxidant activity assays of the study demonstrated that the EPS is capable of scavenging DPPH and O_2_·^–^ radicals. Mathivanan et al. (2021) found that EPS from *L. fusiformis* KMNTT-10 exhibited good antioxidant activity in scavenging DPPH, ABTS, and NO^·^ radicals. In a study conducted by Sánchez-León and et al. (2023), structural and biochemical analyses revealed that the EPS from *B. amyloliquefaciens* RT7 is a heteropolysaccharide composed of various monosaccharides, including mannose, glucose, galactose, and xylose, with an average molecular weight of 7.0794 × 10^4^ Da. The in vitro activity assays of the study revealed that the EPS has antioxidant capacity to scavenge DPPH, OH, and O_2_·^–^ radicals. Moreover, the experiments displayed that EPS could protect HeLa cells against ROS generated with H_2_O_2_ exposure.

### Phenolics, alkaloids, polyketides, macrolides, and extracts

In the literature, it has been documented that members of the domain *Bacteria* also can produce other molecules with antioxidant activity, including phenols, alkaloids, polyketides, and macrolides (Table 6). For example, some studies have examined the antioxidant potential of the phylum *Actinobacteria*. In a similar study, Abdelmohsen et al. (2012) investigated the antioxidant potential of diazepinomicin, a dibenzodiazepine alkaloid derived from *Micromonospora* sp. RV115. They found that diazepinomicin exhibits strong antioxidant potential, as demonstrated by its ferric-reducing power assay, and can protect human kidney and human promyelocytic cells against H_2_O_2_-induced damage by inhibiting ROS accumulation and cell death. In a previous study, Saravana Kumar et al. (2014) found that the ethyl acetate extract prepared from the culture broth of *Streptomyces lavendulae* SCA5 exhibited scavenging activities against ·OH, O_2_·^–^, DPPH, and NO^·^ radicals. They also revealed that the extract has an anticancer effect on the A549 adenocarcinoma lung cancer cell line. Based on the data from GC–MS analysis, the researchers identified the phenolic metabolite as actinomycin C2. In another study, Janardhan et al. (2014) investigated the biological activities of an ethyl acetate extract prepared from the culture broth of the actinomycete *Nocardiopsis alba.* The results from DPPH radical-scavenging and ferric-reducing power measurements indicated that the fraction (F2) exhibited promising antioxidant activity. According to FTIR, NMR, and mass spectrometry analyses, the main bioactive compound was inferred to be (Z)-1-((1-hydroxypenta-2,4-dien-1-yl)oxy)anthracene-9,10-dione. A different research team (Ramalingam and Rajaram 2016) studied the antioxidant activity of 1-hydroxy-1-norresistomycin (HNM) from the actinobacterium *S. variabilis* KP149559 and evaluated its toxicity in zebrafish. Based on DPPH, H_2_O_2,_ and ·OH radicals-scavenging assays, HNM was proven to possess an in vitro antioxidative potential. The toxicity tests revealed that HNM did not cause detrimental effects in the major organs of zebrafish, including the heart, liver, kidney, intestine, and gills. Dholakiya et al. (2017) investigated the antioxidant activity of an ethyl acetate extract prepared from the culture broth of *S. variabilis* RD-5, a bacterium isolated from sea sediment. They found that the extract was capable of scavenging DPPH and H_2_O_2_ radicals, as well as chelating metal ions. Tan and coworkers (2017) reported that the methanolic extract from *Streptomyces *sp. MUM212 demonstrated notable antioxidant potency in DPPH, ABTS, and O_2_·^–^ radical scavenging, as well as metal-chelating activity assays. Furthermore, they reported that the extract inhibited lipid peroxidation and exhibited a capacity to protect Vero cells against H_2_O_2_-induced oxidative damage. They suggested that the antioxidative potential of the extract was related to the total phenolic content; however, other constituents, such as hydrocarbons, alcohols, and cyclic dipeptides, might have contributed to the overall antioxidant ability. Indupalli et al. (2018) investigated the biological activities of the three phenolic secondary metabolites purified from the actinobacterium *Saccharomonospora oceani* VJDS-3. The metabolites were identified as methoxy ethyl cinnamate (ethyl(E)-3-(4-methoxyphenyl)acrylate) (R1), 4-hydroxy methyl cinnamate (methyl(E)-3-(4-hydroxyphenyl)acrylate) (R2), and 4-methylbenzoic acid (R3). They found that the metabolite R3 had antioxidant capacity against DPPH and ABTS radicals, while R1 exhibited characteristic antidiabetic and antiobesity activities. In a previous study, Tan and coworkers (2019) investigated the antioxidant and UV-protective properties of a methanolic extract prepared from the culture broth of *Streptomyces* sp. MUM273b. The authors found that the extract contained phenolics, pyrrole, pyrazine, esters, and cyclic dipeptides, and displayed strong antioxidant potential in radical-scavenging (DPPH, ABTS, and O_2_·^–^), metal-chelating, and lipid peroxidation inhibition assays. Furthermore, they determined that the extract could protect HaCaT keratinocytes against the UVB-induced cell death. Overall, the authors reported a relationship between the antioxidant activities and the total phenolic content of the extract. In another study (Alvariño et al. 2019), a macrolide metabolite, caniferolide A, from the marine actinomycete *S. caniferus*, was found to decrease LPS-induced neuroinflammatory markers in BV2 microglial cells, prevent NF-ΚB-P65 translocation to the nucleus, and activate the Nrf2 pathway. The metabolite reduced the levels of pro-inflammatory cytokines (IL-1β, IL-6, and TNF-α), ROS, and NO^·^, inhibited the activities of iNOS, JNK, and p38, and also blocked BACE1 activity, thereby attenuating Aβ-activation of microglia by drastically reducing ROS levels. Furthermore, the metabolite was ascertained to protect SH-SY5Y cells against H_2_O_2_ toxicity by decreasing ROS levels and increasing GSH content. Considering these results, the authors concluded that caniferolide A can be used as a drug in treating Alzheimer's disease. Kemung et al. (2020) demonstrated that the methanol extract from *Streptomyces* sp. strain MUSC14 exhibited strong antioxidant potential in radical-scavenging assays using ABTS and DPPH radicals, as well as in metal-chelating activity assays. They reported a high correlation between the total phenolic content of the extract and its antioxidant potential. Siddharth et al. (2020) demonstrated that the crude extracts of two actinobacterial strains, *Nocariopsis* sp. SCA 11 and *Nocardioides* sp. SCA 13 exhibited potent scavenging activities towards DPPH and ABTS radicals, as well as antimicrobial effectiveness against pathogenic Gram-positive MRSA bacteria, several Gram-negative bacteria, and fungi. Lee et al. (2020a) examined the anti-aging and antioxidant properties of sarmentosamide, a hexadienamide derivative alkaloid isolated from the marine *Streptomyces *sp. APmarine042. Notably, sarmentosamide decreased UVB-induced matrix metalloproteinase-1 (MMP-1) expression and tumor TNF-α levels in normal human dermal fibroblasts (NHDFs). Additionally, sarmentosamide showed scavenging activity against DPPH radical. In a different study (Gegunde et al. 2021), the researchers examined the anti-inflammatory and antioxidant activities of anhydroexfoliamycin (a secondary metabolite from *Streptomyces*) on microglia of BV2 cells, and they found that the compound inhibited the proinflammatory pathways and reduced the level of intracellular ROS, NO^·^, interleukin 6, and TNF-α. Furthermore, they revealed that the compound had the potential to facilitate the translocation of the erythroid 2-related factor 2 (ERF2) factor and to protect SH-SY5Y cells against neurotoxic mediators. Overall, they informed that anhydroexfoliamycin can be a promising bioactive compound in treating microglia-driven inflammation. In a previous study (Agour et al. 2022), the two compounds (C1 and C2) purified from the fermentation culture of *Actinomyces* spp. were structurally analyzed and subsequently evaluated for their potential antioxidant activity. C1 and C2 were identified as umbelliferone and 1-methoxy-3-methyl-8-hydroxyanthraquinone, respectively. The in vitro antioxidant activity assays revealed that both compounds, notably C2, exhibited high potency in scavenging DPPH radicals. Djebbah and et al. (2022) focused on assessing the antibacterial and antioxidant capacities of an extract prepared from the fermentation culture of *Streptomyces* sp. GLD25. They found that the extract exhibited significant antibacterial activity against *S. aureus* and *B. subtilis*, and displayed promising antioxidant potential in DPPH radical-scavenging and ferric-reducing power assays. GC–MS analysis revealed that the main metabolites of the active extract were diisooctyl phthalate, 6-hydroxyheptanoic acid, hexadecanoic acid, benzeneacetic acid, and 3-(3,5-di-tert-butyl-4-hydroxyphenyl)propionic acid. Thayanuwadtanawong et al. (2023) evaluated the in vitro antioxidant activities of nine compounds (compounds 1–9) purified from the ethyl acetate extract of the terrestrial actinobacterium *S. telluris* sp. Based on the results from the DPPH radical-scavenging assay, they determined that the phenolic compound 7 (3,4-dihydroxybenzaldehyde) had a strong antioxidant capacity equal to that of ascorbic acid. A recent study performed by Weslati and co-workers (2023) elucidated that the crude extract from *Streptomyces* sp. The FR7 strain exhibited strong DPPH radical-scavenging activity and protected yeast cells from H_2_O_2_-induced oxidative stress. The study also demonstrated that the extract exhibited significant antimicrobial activity against various bacterial pathogens. The strong activities were mainly attributed to the presence of the polyketides with methylsalicylic acid moiety in the extract. In another study (Mangamuri et al. 2022), five active compounds (C1–C5) were purified from the ethyl acetate extract prepared from the culture filtrate of the actinobacterium *S. arabica* VSM-25. Among the purified compounds, C1 (indole-3-carboxaldehyde) was found to possess a higher inhibitory effect against pathogenic bacteria and fungi, while the compound C5 (3,4-dihydroxybenzaldehyde) achieved the highest in vitro antioxidant activities in DPPH and NO· radicals-scavenging assays.

**Table 6:** Bacteria-derived antioxidant phenolics, alkaloids, polyketids, macrolides and extracts

Compound	Bacterium/phylum	Activity	References
Diazepinomicin (an alkaloid)	*Micromonospora* sp. RV115 (*Actinobacteria*)	Ferric reducing power activity as well as the prevention of ROS accumulation in human promyelocytic cell line HL-60 exposed to H_2_O_2_ (In vitro)	(Abdelmohsen et al. 2012)
Anhydroexfoliamycin	*Streptomyces* (*Actinobacteria*)	Anti-inflammatory (the reduction of NO^·^, interleukin 6, and TNF-α levels) and antioxidant (the reduction of intracellular ROS in microglia BV2 cells) activities (In vitro)	(Gegunde et al. 2021)
Methanol extract containing phenolics, hydrocarbons, alcohols and cyclic dipeptides	*Streptomyces* sp. MUM212 (*Actinobacteria*)	DPPH, ABTS and O_2_^·–^ radicals-scavenging and metal-chelating activities (22.03%, 61.52%, 37.47%, and 41.98% at a concentration 4 mg/mL, respectively). Besides, lipid peroxidation inhibition activity (16.72% at a concentration 4 mg/mL in Vero cells (In vitro)	(Tan et al. 2017)
Methanol extract containing phenolics, pyrrole, pyrazine, ester, and cyclic dipeptides	*Streptomyces* sp. MUM273b (*Actinobacteria*)	DPPH, ABTS and O_2_^·–^ radicals-scavenging, metal- chelating and lipid peroxidation inhibition activities (8.83%, 56.24%, 22.47%, 23.44% and 25.88% at a concentration of 4 mg/mL, respectively). Furthermore, the ability to protect HaCaT keratinocytes against UVB-induced cytotoxicity (In vitro)	(Tan et al. 2019)
Methanolic extract containing phenolics	*Streptomyces* sp. MUSC14 (*Actinobacteria*)	Scavenging activities against ABTS (62.72% at a concentration of 4 mg/mL) and DPPH (28.14% and 24.71% at the concentrations of 2 and 4 mg/mL, respectively) as well as metal- chelating activity (55.82% at a concentration of 4 mg/mL) (In vitro)	(Kemung et al. 2020)
Ethyl acetate extract	*Streptomyces* sp. GLD25 (*Actinobacteria*)	DPPH scavenging (IC_50_ values of 20.12 and 0.083 mg/mL for extract and AA, respectively) and ferric reducing power (IC_50_ values of 8.80 and 0.03 mg/mL for the extract and AA, respectively) activities as well as antibacterial effectiveness towards *S. aureus* and *B. subtilis* (In vitro)	(Djebbah et al. 2022)
Polyketides	*Streptomyces* sp. FR7 (*Actinobacteria*)	DPPH (IC50 = 1.3 μg mL) scavenging activity, protection of yeast cells from H_2_O_2_-induced oxidative stress, and the antibacterial activities against *M. luteus*, *S. aureus*, *L. monocytogenes,* and *P. aeruginosa* (In vitro)	(Weslati et al. 2023)
Sarmentosamide (an alkaloid)	*Streptomyces* sp. APmarine042 (*Actinobacteria*)	DPPH radicals-scavenging activity (35–40% at a concentration of 2 mg/mL) and the ability to protect human dermal fibroblasts (NHDF) against UVB (In vitro)	(Lee et al. 2020a)
1-hydroxy-1-norresistomycin	*S. variabilis* KP149559 (*Actinobacteria*)	DPPH, H_2_O_2_ and ·OH radicals-scavenging activities (respectively IC50 values of 300, 400 and 300 μg /mL of the metabolite). Besides, antibacterial activity against *E. coli, V. cholerae, K. pneumoniae, P. aeruginosa, Enterobacter sp.* and *Streptococcus* sp. (In vitro)	(Ramalingam and Rajaram 2016)
Ethyl acetate extract	*S. variabilis* RD-5 (*Actinobacteria*)	DPPH (82.86% at a concentration of 5.0 mg/mL) and H_2_O_2_ (74.5% at a concentration of 0.05 mg/mL) scavenging as well as metal chelating power (89% at a concentration of 5.0 mg/mL) (In vitro)	(Dholakiya et al. 2017)
Extract containing actinomycin C2 (a phenolic compound)	*S. lavendulae* SCA5 (*Actinobacteria*)	Scavenging activities against ·OH (IC_50_ 617.84 μg/mL), DPPH (IC_50_ 507.61 μg/mL), O_2_^·–^ (IC_50_ 864.71 μg/mL) and NO^·^ (IC_50_ 730.92 μg/mL). Besides, lipid peroxidation inhibition activity (50% at a concentration of 838.83 μg/mL) and anticancer effectiveness against A549 cell line (In vitro)	(Saravana Kumar et al. 2014)
Caniferolide A (a macrolide)	*S. caniferus* (*Actinobacteria*)	Reduction in levels of ROS, NO^·^, pro-inflammatory cytokines (IL-1β, IL-6, and TNF-α) in BV2 microglial cells exposed to LPS (In vitro)	(Alvariño et al. 2019)
3,4-dihydroxybenzaldehyde ((C7, a phenolic compound)	*S. telluris* sp. Nov (*Actinobacteria*)	DPPH radicals scavenging activity (IC_50_ values of 17.4, 17.7 and 59.0 µg/mL for C7, AA and BHT, respectively) (In vitro)	(Thayanuwadtanawong et al. 2023)
Umbelliferone (C1) and 1-methoxy-3-methyl-8-hydroxy-anthraquinone (C2)	*Actinomyces* spp. AW6 (*Actinobacteria*)	DPPH radicals-scavenging activity (30.20%, 55.25% and 78.50% for C1, C2 and AA, respectively). Besides, antibacterial effectiveness of C1 and C2 towards *E. coli* and *S. aureus* (In vitro)	(Agour et al. 2022)
Indole-3-carboxaldehyde (C1), 2, 3-dihydroxy benzoic acid (C2), vanillic acid (C3), daidzein (C4), and 3, 4-Dihydroxy benzaldehyde (C5)	*Streptomonospora arabica* VSM-25 (*Actinobacteria*)	Scavenging activities against DPPH (59.87%, 61.625%, 52.153%, 34.217%, 64.562% and 72.50% for C1, C2, C3, C4, C5 and AA at 400 μg/mL, respectively) and NO^·^ (66.22%, 62.12%, 58.16%, 70.66% and 79.22% for C1, C2, C3, C5 and AA at 400 μg/mL, respectively). Besides, antibacterial, antifungal and anticancer activities (In vitro)	(Mangamuri et al. 2022)
(Z)-1-((1-hydroxypenta-2,4-dien-1-yl)oxy)anthracene-9,10-dione	*Nocardiopsis alba* (*Actinobacteria*)	DPPH radical scavenging (greater than 50% at concentration of 50 *μ*g/mL) and ferric reducing power activities (In vitro)	(Janardhan et al. 2014)
4-methyl benzoic acid	*Saccharomonospora oceani* VJDS-3 (*Actinobacteria*)	DPPH and ABTS scavenging activities (73.08% and 99.74% at the concentrations of 25 µg/mL and 50 µg/mL, respectively) as well as antidiabetic and antiobesity activities (In vitro)	(Indupalli et al. 2018)
Ethyl acetate extracts (SCA 11 and SCA 13)	*Nocariopsis* sp. SCA 11 and *Nocardioides* sp. SCA 13 (*Actinobacteria*)	ABTS (IC_50_ values of 48.24, 37.91 and 9.87 µg/mL for SCA11, SCA13 and trolox, respectively) and DPPH (IC_50_ values of 30.91, 42.30 and 11.07 µg/mL for SCA11, SCA13 and trolox, respectively) radicals-scavenging activities as well as antibacterial (against MRSA strains and Gram-negative bacteria) and antifungal effectiveness (In vitro)	(Siddharth et al. 2020)
Methanolic and ethanolic extracts containing phenolics and flavonoids	*Aphanizomenon gracile, Aphanizomenon flos-aquae,* *Nostoc* and *Planktothrix mougeotii* (*Cyanobacteria*)	DPPH scavenging (between 8.8% and 10.7%, corresponding to 17.3 μg/mL to 20.7 μg/mL as Trolox equivalents) and lipid peroxidation inhibition (In vitro)	(Guerreiro et al. 2020)
Ethanol extracts (quinic acid, gallic acid, chlorogenic acid, catechin, epicatechin, kaempferol, rutin and apiin)	*Nostoc* and *Arthrospira* strains (*Cyanobacteria*)	DPPH scavenging (IC_50_ value: 0.04–9.47 mg/mL for the extracts and 9.8 μg/mL for BHT) and ferric reducing power (8.36–21.01 mg AAE/g for extracts and 25.32 mg AAE/g for BHT) (In vitro)	(Blagojević et al. 2018)
Methanol extract containing polyphenolics (gallic, chlorogenic, caffeic, vanillic and ferulic acids) and flavonoids (rutin, quercetin and kaempferol)	*Anabaena constricta* and other *Cyanobacteria* strains	DPPH and ABTS scavenging, deoxyribose protection and ferric reducing power activities (IC_50_ values: 0.91 mg/mL, 0.23 mg/mL, 0.63 mg/mL and 0.9 mg/mL, respectively) (In vitro)	(Singh et al. 2017b)
Methanol-chloroform extracts containing dibutyl phthalate, hexadecanoic acid and 1, 2-benzene dicarboxylic acid	*Oscillatoria* sp. SSCM01 and *Phormidium* sp. SSCM02 (*Cyanobacteria*)	DPPH radical scavenging activity (48% at 60 a concentration of μg/mL concentration) and antimicrobial effects (against *S. aureus, S. typhi,* and *C. albicans*) (In vitro)	(Nainangu et al. 2020)
Scytonemin (indole-alkaloid)	*Leptolyngbya mycodia* and *Phormidium* sp (*Cyanobacteria*)	DPPH radical-scavenging activity (48.84% at a concentration of 200 μg/L) (In vitro)	(Madrahi and Naeimpoor 2022)
Methanol and acetone extracts containing phenolics and flavonoids	*Lyngbya majuscula* SB12-13 and *L. martensiana* SBD24) (*Cyanobacteria*)	DPPH (IC_50_ values of methanol and ethanol extracts: 251.34 and 306.04 µg/mL for SB12-13, and 257 and 339.11 µg/mL for SBD24, respectively), ABTS (IC_50_ values of the methanol and ethanol extracts: 282.24 and 310.61 µg/mL for SB12-13 and 326.69 and 353.03 µg/mL for SBD24, respectively) and ferric reducing power (the highest value of 0.762 µg/mL for methanol extracts of SB12-13). Besides, antimicrobial activites of the extracts against bacterial and fungal pathogens (In vitro)	(Dash et al. 2023)

*ABTS* 2,2'-azino-bis(3-ethylbenzothiazoline-6-sulfonic acid, *DPPH* 2,2-Diphenyl-1-picrylhydrazyl, *ROS* reactive oxygen species, ·OH, hydroxyl radical, O_2_^·–^ superoxide radical, *H*_*2*_*O*_*2*_ hydrogen peroxide and *NO*^·^ nitric oxide radical. *BHT* Butylated hydroxytoluene and *AA* ascorbic acid represent the control compounds used in antioxidant activity assays

In addition to *Actinobacteria*, members of other phyla, including *Cyanobacteria*, *Proteobacteria*, *Bacteroidetes*, and *Firmicutes*, also produce various phenolic compounds and alkaloids. For instance, Guerreiro et al. (2020) evaluated the antioxidant properties of methanol and ethanol extracts from the lyophilized biomass of different cyanobacterial strains isolated from freshwater and wastewater habitats. They determined that among the tested strains, four strains (*Aphanizomenon gracile*, *A. flos-aquae, Nostoc,* and *Planktothrix mougeotii)* showed higher antioxidant activities in DPPH and β-carotene bleaching measurements. Furthermore, they found that most cyanobacterial extracts can protect HEK293T cells against H_2_O_2_-induced cytotoxicity. A previous study by Blagojević and et al. (2018) focused on investigating the phenolic content and antioxidant capacity of ethanol extracts from ten cyanobacterial strains. The chemical analyses revealed the presence of various phenolics in the extracts, including quinic acid, gallic acid, and chlorogenic acid. The activity measurements showed that the extract of *Nostoc *LC1 B exhibited the highest scavenging potential against the DPPH radical. In contrast, the extracts of *Arthrospira* strains demonstrated higher ferric-reducing power activity. In a previous study (Singh et al. 2017b), the chemical composition and biological activities of methanol extracts from twenty terrestrial cyanobacteria were investigated. The extracts were ascertained to include various polyphenolics and flavonoids. Among all the extracts tested, that of *Anabaena constricta* was found to have the highest antioxidant capacity, as indicated by the results of DPPH and ABTS radical-scavenging assays, ferric reducing power, and deoxyribose protection assays. A previous study (Nainangu et al. 2020) evaluated the biological activities (antimicrobial and antioxidant) and toxicity profiles of the methanol-chloroform extracts from two freshwater filamentous cyanobacteria (*Oscillatoria* sp. SSCM01 and *Phormidium* sp. SSCM02). The extract of SSCM01 was shown to have a higher antioxidant capacity, as measured in the DPPH radical-scavenging assay, and antibacterial effectiveness. This extract was determined to contain several metabolites, including hexadecanoic acid, 1,2-benzene dicarboxylic acid, and dibutyl phthalate. Another research team (Madrahi and Naeimpoor 2022) reported that an indole-alkaloid, scytonemin, from two filamentous cyanobacteria, *Leptolyngbya mycodia* and *Phormidium* sp., exhibited strong potential for scavenging DPPH radicals. A recent study by Dash and co-workers (2024) focused on examining the biological activities of methanol and acetone extracts of two species of *Cyanobacteria* (*Lyngbya majuscula* SB12-13 and *L. martensiana* SBD24). The extracts were determined to be rich in total phenolics and flavonoids. The in vitro antioxidant activity assays revealed that extracts of *L. majuscula* have the highest radical-scavenging activity (against DPPH and ABTS) and ferric-reducing power potential. Furthermore, the extracts of *L. majuscula* exhibited a higher antibacterial effect than those of *L. martensiana* SBD24.

## Potential applications and limitations of bacteria-derived antioxidants

In studies conducted to date, conflicting results have been obtained regarding the effectiveness of synthetic or natural compounds with antioxidant activity in combating oxidative stress-induced diseases. For example, some in vivo animal and preclinic human studies have reported that the antioxidants (glutathione, carvacrol, resveratrol, astaxanthin, CoQ10) from natural sources (plants, fungi, and algae) can show therapeutic effectiveness towards oxidative stress-related pathologies (Xu et al. 2018; Bahramrezaie et al. 2019; Tanzilli et al. 2019; El-Baz et al. 2023; Dost et al. 2024). On the contrary, some studies have reported that the natural antioxidants do not exhibit a protective role against these pathologies in the in vivo animal and human studies (Goodman et al. 2011; Bjelakovic et al. 2012; Ochiai et al. 2019; Forman and Zhang 2021; Pappolla et al. 2024). In short, there are conflicting findings regarding the therapeutic effectiveness of natural or synthetic antioxidants in combating oxidative stress-induced diseases.

It has been hypothesized that a significant limitation of using antioxidants in the treatment of oxidative stress-induced diseases is the obstacle in delivering an adequate amount of antioxidants to the targeted intracellular location, for instance, inside mitochondria (Murphy 2014; Banerjee et al. 2022). At this point, using conjugates to transport antioxidants into targeted organs or intercellular locations is considered a promising approach. For example, these conjugates can be prepared by combining the antioxidants with the triphenylphosphonium (TPP^+^), which can pass the blood–brain barrier and target mitochondria (Wang et al. 2020). The preclinical studies performed have shown that oral or intraperitoneal administration of these conjugates (mitochondria-targeted antioxidants combined with TPP +) provides better protection against Parkinson’s disease compared to non-targeted ones (Yang et al. 2009; Ghosh et al. 2012; Ramis et al. 2015). Similarly, MitoQ10, a mitochondria-targeted antioxidant, has been reported to exhibit protection against cardiovascular diseases and infertility in preclinical studies (McLachlan et al. 2014; Ding et al. 2019).

Although the effectiveness of antioxidant molecules has not been fully proven in preclinical studies, research focusing on new antioxidant molecules and their therapeutic potential continues to attract interest. For instance, as seen in Tables 1, 2, 3, 4, 5, 6, numerous literature studies focus on the antioxidant activities of currently known metabolites and newly discovered ones derived from bacteria. However, the data summarized in these tables indicate that the antioxidant effectiveness of bacteria-derived metabolites has been primarily tested through in *vitro* activity assays and in some in vivo animal studies. At this point, it is possible to say that achieving equal success may not be feasible in human models. Therefore, further animal and human studies should be conducted to elucidate the effectiveness of bacteria-derived antioxidants in combating oxidative stress-induced diseases. If their effectiveness is proven, they may be used in clinic to prevent or treat the oxidative stress-induced pathologies such as skin aging, cancer, neurodegenerative diseases, cardiovascular diseases, diabetes, infertility, retinal diseases, Crohn’s disease, rheumatoid arthritis and inflammatory bowel diseases (Fig. 4). For example, Tan et al. (2017) found that *Streptomyces*-derived methanol extract exhibited radical-scavenging activity and inhibited lipid peroxidation in kidney Vero cells. This finding suggests that the extract or its bioactive components may be evaluated for their potential to prevent oxidative stress-induced kidney pathologies. Alvariño et al. (2019) revealed that caniferolide A from *S. caniferus* reduced ROS and NO^·^ levels in BV2 microglial cells. Based on this finding, it is possible to say that caniferolide A may be a drug candidate for the treatment or prevention of oxidative stress-mediated neurodegenerative diseases. Furthermore, it is well known that natural metabolites with antioxidant activity also exhibit skin anti-aging properties by preventing ROS accumulation induced by intrinsic or extrinsic factors, such as chemicals and UV damage (Kageyama and Waditee-Sirisattha 2019; Ahmed et al. 2020; Bouzroud et al. 2023; Feng et al. 2024). For example, Lee et al. (2021) demonstrated that the EPS from the bacterium *L. plantarum* HY7714 reduced UVB-induced cytotoxicity and ROS accumulation in HS68 (CRL-1635) human dermal fibroblasts, thereby exhibiting anti-aging activity in the skin. Zheng et al. (2022) reported that the extract with antioxidant activity from the bacterium *S. chattanoogensis* THA-663 (THA-663S) can prevent extracellular matrix degradation, promote procollagen type I synthesis, and reduce ROS generation in UVB-irradiated HaCaT cells, thereby displaying anti-aging potency. In a separate study (Lee et al. 2020b), researchers found that prodigiosin from the bacterium *Hahella chejuensis* decreased ROS accumulation and enhanced collagen synthesis in HaCaT human skin keratinocytes exposed to UV irradiation. Furthermore, they found that the prodigiosin was not cytotoxic and could improve the proliferation of HaCaT keratinocytes. Due to these potential activities, the prodigiosin was considered to have skin anti-aging effects. A different research group (Suryawanshi et al. 2015) found that the bacterial pigments prodigiosin and violacein displayed antioxidant activity and also enhanced the photoprotective effectiveness of commercial sunscreens. Considering that the antioxidant metabolites also have the potency to protect skin cells against various stress factors, it is possible to say that bacteria-derived antioxidant molecules may find applications in the cosmetic industry for the preparation of anti-aging formulations, such as sunscreen creams.

Moreover, a literature survey revealed that bacteria-derived metabolites or extracts with antioxidant activity also exhibit other bioactive properties, such as antimicrobial, anti-inflammatory, anticancer, anti-obesity, antiviral, and wound-healing activities (Tables 1–6). For example, some compounds (polysaccharides, peptides and peptides) and extracts with antioxidant activity were determined to have also anti-diabietic and antimicrobial (against various pathogen bacteria and fungi) activities (Ayed et al. 2015; Suryawanshi et al. 2015; Jemil et al. 2017; Elnahas et al. 2017; Sonani et al. 2017; Ayyash et al. 2020; Siddharth et al. 2020; Jaber et al. 2021; Saleem et al. 2021; Djebbah et al. 2022; Gurkok 2022; Mangamuri et al. 2022; Metwally et al. 2022; Sudhakar et al. 2022; Yu et al. 2022; Soni et al. 2023; Mohamed et al. 2023; Naik and Gupte 2024; Suphan et al. 2023; Priyanka et al. 2024). Based on this finding, it is possible to say that, thanks to their antimicrobial activities, bacterial antioxidant molecules or extracts may also be used in the prevention of infectious diseases, the preparation of pathogen-free foods, and the extension of the shelf life of these foods. Furthermore, due to their anti-diabetic and anticancer activities, they may find applications in the nutraceutical and pharmaceutical industries.

## Conclusion and future perspectives

The excessive accumulation of reactive oxygen (ROS) and nitrogen species (RNS) in the cells causes oxidative stress and is linked to various human health conditions. Both endogenous and exogenous antioxidants synergistically eliminate excess ROS and RNS, thereby protecting the body against oxidative stress-induced pathologies. Humans obtain exogenous antioxidants mainly from plants through diet or supplements. However, we can also get them from other natural sources. This review reveals that many exogenous antioxidant metabolites, including polysaccharides, pigments, phycobiliproteins, mycosporins-like amino acids, peptides, phenolics, alkaloids, and polyketides, can be obtained from five phyla of the domain *Bacteria*: *Actinobacteria*, *Cyanobacteria*, *Bacteroidetes*, *Firmicutes,* and *Proteobacteria.* Some of these antioxidants (phycobiliproteins, prodigiosin, and violacein) cannot be synthesized by other organisms, such as plants and fungi, and their sole source is bacteria. Bacteria-derived metabolites and extracts display the in vitro and in vivo antioxidant activities. However, there are some gaps in the literature regarding bacteria-derived antioxidant compounds. The first one is that bacteria-derived peptides and phycobiliproteins are less studied when compared to other bacterial metabolites. The second is that there are fewer studies on the antioxidant metabolites of the phylum *Bacteroidetes* when compared to different phyla. The third gap is that the antioxidant potential of bacteria-derived metabolites has been tested primarily in in vitro assays, and there are limited studies on in vivo models. The fourth gap in the literature is that the therapeutic potential of bacteria-derived antioxidants in addressing oxidative stress-induced pathologies has not been investigated yet. Therefore, we suggest that bacteria-derived antioxidant metabolites and extracts should be tested in more in vivo and preclinical studies. Furthermore, it is possible to say that the therapeutic effectiveness of bacteria-derived antioxidants in combating oxidative stress-induced diseases can be improved if they are modified with molecules such as triphenylphosphonium, which target specific organs or mitochondria. If their safety profile and efficacy are proven in in vivo and clinical studies, bacterial antioxidants can be used in the cosmetic industry as an anti-aging agent for the skin and in medicine as a drug or supplement against oxidative stress-induced pathologies.
